# The appropriate nutrient conditions for methicillin-resistant *Staphylococcus aureus* and *Candida albicans* dual-species biofilm formation in vitro

**DOI:** 10.1038/s41598-024-83745-1

**Published:** 2025-01-02

**Authors:** Pavlína Vávrová, Ondřej Janďourek, Adéla Diepoltová, Petr Nachtigal, Klára Konečná

**Affiliations:** Department of Biological and Medical Sciences, Charles University, Faculty of Pharmacy in Hradec Králové, 2089, Zborovská, Hradec Králové, 500 03 Czech Republic

**Keywords:** Microbiology, Bacteria, Biofilms, Fungi

## Abstract

Polymicrobial biofilms, the reason for most chronic wound infections, play a significant role in increasing antibiotic resistance. The in vivo effectiveness of the new anti-biofilm therapy is conditioned by the profound evaluation using appropriate in vitro biofilm models. Since nutrient availability is crucial for in vitro biofilm formation, this study is focused on the impact of four selected cultivation media on the properties of methicillin-resistant *Staphylococcus aureus* and *Candida albicans* dual-species biofilms. To reflect the wound environment, Tryptic soy broth, RPMI 1640 with and without glucose, and Lubbock medium were supplemented with different amounts of host effector molecules present in human plasma or sheep red blood cells. The study demonstrates that the Lubbock medium provided the most appropriate amount of nutrients regarding the biomass structure and the highest degree of tolerance to selected antimicrobials with the evident contribution of the biofilm matrix. Our results allow the rational employment of nutrition conditions within methicillin-resistant *Staphylococcus aureus* and *Candida albicans* dual-species biofilm formation in vitro for preclinical research. Additionally, one of the potential targets of a complex antibiofilm strategy, carbohydrates, was revealed since they are prevailing molecules in the matrices regardless of the cultivation media.

**Supplementary Information:**

The online version contains supplementary material available at 10.1038/s41598-024-83745-1.

## Introduction

Antimicrobial resistance (AMR) is a rapidly increasing threat worldwide. According to World Health Organisation statements, it has been declared one of the top 10 global public health threats humanity is facing^1^. A significant force in driving AMR is the microbial ability to create multicellular consortia called biofilms^2^. The coexistence of microorganisms in communities represents the predominant lifeform in vivo and brings many advantages to microbial participants^3^.

Generally, microbial biofilms are recognised as three-dimensional complex communities of microorganisms embedded in a biofilm matrix composed of extracellular polymeric substances (EPS) consisting mainly of polysaccharides, proteins, and extracellular DNA (eDNA)^4^. EPS are responsible for the structural and functional properties of biofilms, including the whole consortium’s existence and virulence^4,5^. In addition, the biofilm matrix represents a protective barrier for the biofilm-forming microbial agents. It plays a crucial role in the ability of participants to withstand hostile conditions, including exposure to antimicrobial drugs. It is stated that microorganisms present in biofilms can resist 10 to 1000-fold higher doses of antimicrobials compared to the same microbial agents present in planktonic form^5^.

Microbial biofilms have been recognised as being involved in many clinical infections and are responsible for the majority (up to 80%) of all chronic infections^6^. The most naturally occurring microbial biofilms, including commensal biofilms in humans, are mixed-species consortia^7^. Infections associated with multi-species biofilms lead to more severe symptoms and significantly higher mortality compared to infections caused by individual pathogens^7–9^. Generally, multi-species microbial biofilm communities show complex synergic inter-species or inter-kingdom interactions. These circumstances have a positive final impact on the multiplication ratio, virulence, and AMR of participants and their production of EPS^4,9,10^.

*Staphylococcus aureus* (SA) and *Candida albicans* (CA) are commensal microorganisms that predominantly colonize the skin and mucous membranes in most humans in coexistence. However, as opportunistic pathogens, these microbial agents can cause both superficial and life-threatening systemic infections, especially in immunocompromised patients. It has been demonstrated that these agents can be present in biofilm-associated infections. The mutual interaction of SA and CA within mixed infection is recognized as one of the best examples of “lethal synergism”. This is defined as excess mortality compared to otherwise “survivable” monomicrobial infections^11–13^. Wound infections belong to the most clinically significant biofilm-associated infections caused by these pathogens. The susceptibility of a wound to this opportunistic pathogen colonization is related to specific environmental conditions, including the presence of wound exudate. Biofilms have been reported as an underlying reason for the non-healing and prolonged infections observed in most (if not all) chronic wounds^14–16^.

Indeed, biofilm-related infections, and especially those that are associated with polymicrobial inter-kingdom biofilm consortia, represent a particularly challenging problem^11^. Thus, effective anti-biofilm therapeutic strategies are critically needed.

One of the first steps in the discovering of new anti-biofilm-acting drugs and strategies is based on experiments that evaluate effectiveness against preformed microbial biofilm consortia in vitro^17–19^. This decisive phase should not be neglected. Only a thorough evaluation of anti-biofilm activities on adequate biofilm models can help to prevent obtaining falsely promising results and limit the amount of failure in follow-up, advanced preclinical, and clinical studies^17,19,20^.

The goal of this research was to determine optimal nutritional conditions reflecting the moist environment in wounds leading to the formation of a methicillin-resistant SA (MRSA) and CA dual-species biofilm model in vitro. The required attributes were a high degree of tolerance to antimicrobial drugs with an evident contribution of the biofilm matrix. Concerning the employment of this model especially for preclinical screening of anti-biofilm activities, the emphasis on reproducibility, controllability, user-friendliness, and cost-effectiveness of the model was also taken into account. The mentioned attributes of biofilm consortia were preferred to reduce falsely promising results in basic research and increase the probability of success in advanced studies.

The study shows that variable nutrient availability leads to the formation of biofilms with different attributes including diverse tolerance to antimicrobial drugs. These findings underline the importance of the methodological settings of the preclinical experiments. Our results therefore allow the rational employment of nutrition conditions within MRSA + CA dual-species biofilm formation in vitro for preclinical research of anti-biofilm strategies.

## Results

### Composition of the cultivation media included in the MRSA + CA dual-species in vitro biofilm formation

Four cultivation media with different nutrient availability were chosen for this study: Tryptic soy broth (TSB), RPMI 1640 (RPMI) with and without glucose (w/o GLU), and the Lubbock medium^21^. The Lubbock medium was slightly modified by using sheep red blood cells instead of horse red blood cells. To obtain the relevant results mimicking in vivo conditions, all chosen media were supplemented with different amounts of human plasma (HP). Two media, namely the Lubbock medium and RPMI w/o GLU were additionally supplemented with freeze-thaw lysed sheep red blood cells (RBC).

As shown in Table S1, the most nitrogen-rich media were the Lubbock medium and RPMI w/o GLU supplemented with 33% (v/v) HP, and 5% (v/v) RBC (RPMI w/o GLU + HP + RBC). The most glucose-rich media in this study were TSB and RPMI, both supplemented with 10% (v/v) HP (TSB + HP; RPMI + HP). For illustration, the reported basic composition of wound exudate is also shown in Table S1.

The reference microbial strains, namely MRSA (ATCC 43300, CCM 4750) and CA (ATCC 90028, CCM 8261) included in previously published studies^22,23^ focused on in vitro mono-species biofilm formation, were employed in this study.

### Impact of the four selected media on the total MRSA and CA mono-species and MRSA + CA dual-species biofilm biomass production

For a quantitative analysis of the MRSA and CA mono- and dual-species total biofilm biomass produced in four different cultivation media after 24 h, the crystal violet (CV) staining method was employed.

**Fig. 1:**
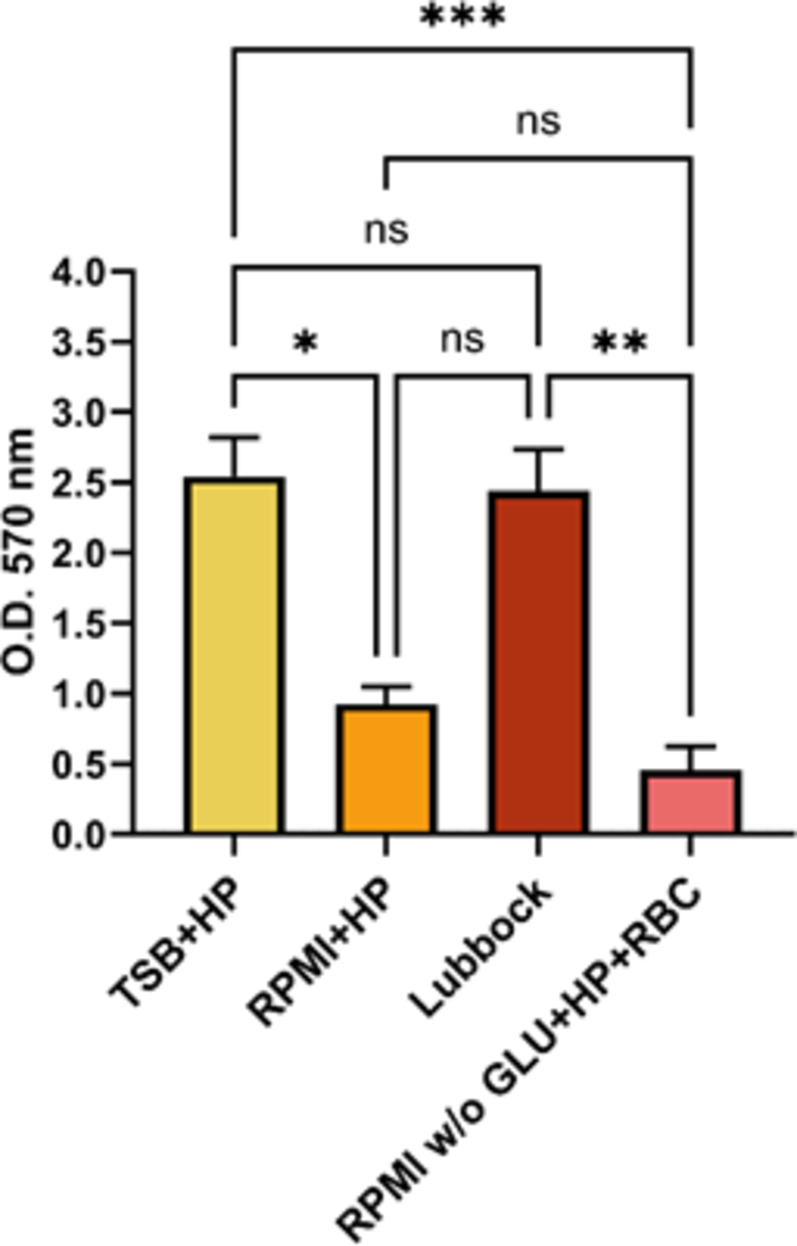
The total biofilm biomass of methicillin-resistant *Staphylococcus aureus* and *Candida albicans* dual-species biofilms. The methicillin-resistant *Staphylococcus aureus* (ATCC 43300) and *Candida albicans* (ATCC 90028) dual-species biofilms were cultivated in vitro in four different cultivation media for 24 h. TSB + HP – Tryptic soy broth + 10% (v/v) human plasma, RPMI + HP – RPMI 1640 + 10% (v/v) human plasma, Lubbock – Bolton broth + 50% (v/v) human plasma + 5% (v/v) sheep red blood cell lysate, RPMI w/o GLU + HP + RBC – RPMI 1640 without glucose + 33% (v/v) human plasma + 5% (v/v) sheep red blood cell lysate. The values represent the mean ± SEM. Data were analyzed using one-way ANOVA, and a *p*-value < 0.05 was accepted as statistically significant. O.D. – optical density, ns – not significant.

The greatest total dual-species biofilm biomass production was achieved in the Lubbock and TSB + HP media. The lowest production dual-species biofilm biomass was revealed in RPMI w/o GLU + HP + RBC medium (see Fig. 1). Similarly, a visual inspection of the 24-well plates after 24-hour cultivation of MRSA + CA biofilms in the four selected media revealed higher biomass in the TSB + HP and the Lubbock media (see Supplementary information, Fig. S1).

It should be mentioned that the total biofilm biomass detected by the CV staining method reflected both the total number of biofilm-forming cells, together with the total quantity of biofilm matrix produced by biofilm consortium participants. The criteria used for the categorization of biofilm producer phenotypes introduced by Stepanović *et al*.^24^ were used for both MRSA and CA strains alone, as well as for the MRSA + CA dual-species consortia.

As shown in Table 1, the most prolific biofilm producer phenotype (moderate biofilm producer) of the MRSA strain was achieved in the TSB + HP and the Lubbock media. The CA strain exhibited the highest biofilm producer phenotype (moderate biofilm producer) in the Lubbock medium. In the dual-species community of MRSA + CA, the greatest biofilm producer phenotype (strong biofilm producer) was recognised after 24-hour biofilm formation in both the TSB + HP and the Lubbock media.

**Table 1:**
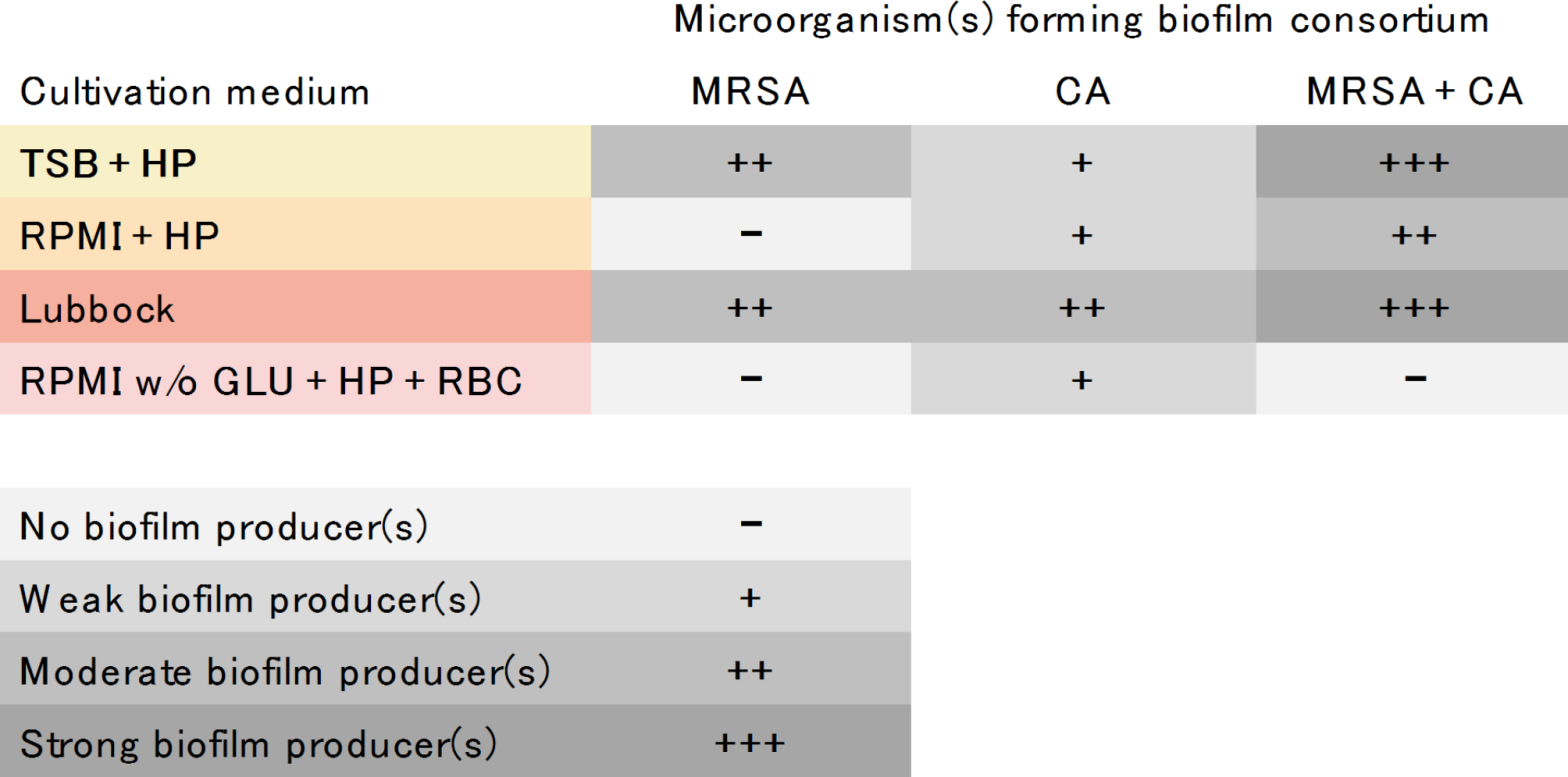
Categorization of the biofilm-producing phenotype of MRSA, CA, and MRSA + CA dual-species consortia, according to the in vitro biofilm-forming capacity after 24 h cultivation in four selected cultivation media.

MRSA – methicillin-resistant *Staphylococcus aureus* (ATCC 43300) in mono-species biofilm consortium, CA – *Candida albicans* (ATCC 90028) in mono-species biofilm consortium, MRSA + CA – methicillin-resistant *Staphylococcus aureus* (ATCC 43300) and *Candida albicans* (ATCC 90028) in dual-species biofilm consortium, TSB + HP – Tryptic soy broth + 10% (v/v) human plasma, RPMI + HP – RPMI 1640 + 10% (v/v) human plasma, Lubbock – Bolton broth + 50% (v/v) human plasma + 5% (v/v) sheep red blood cell lysate, RPMI w/o GLU + HP + RBC – RPMI 1640 without glucose + 33% (v/v) human plasma + 5% (v/v) sheep red blood cell lysate.

**Fig. 2:**
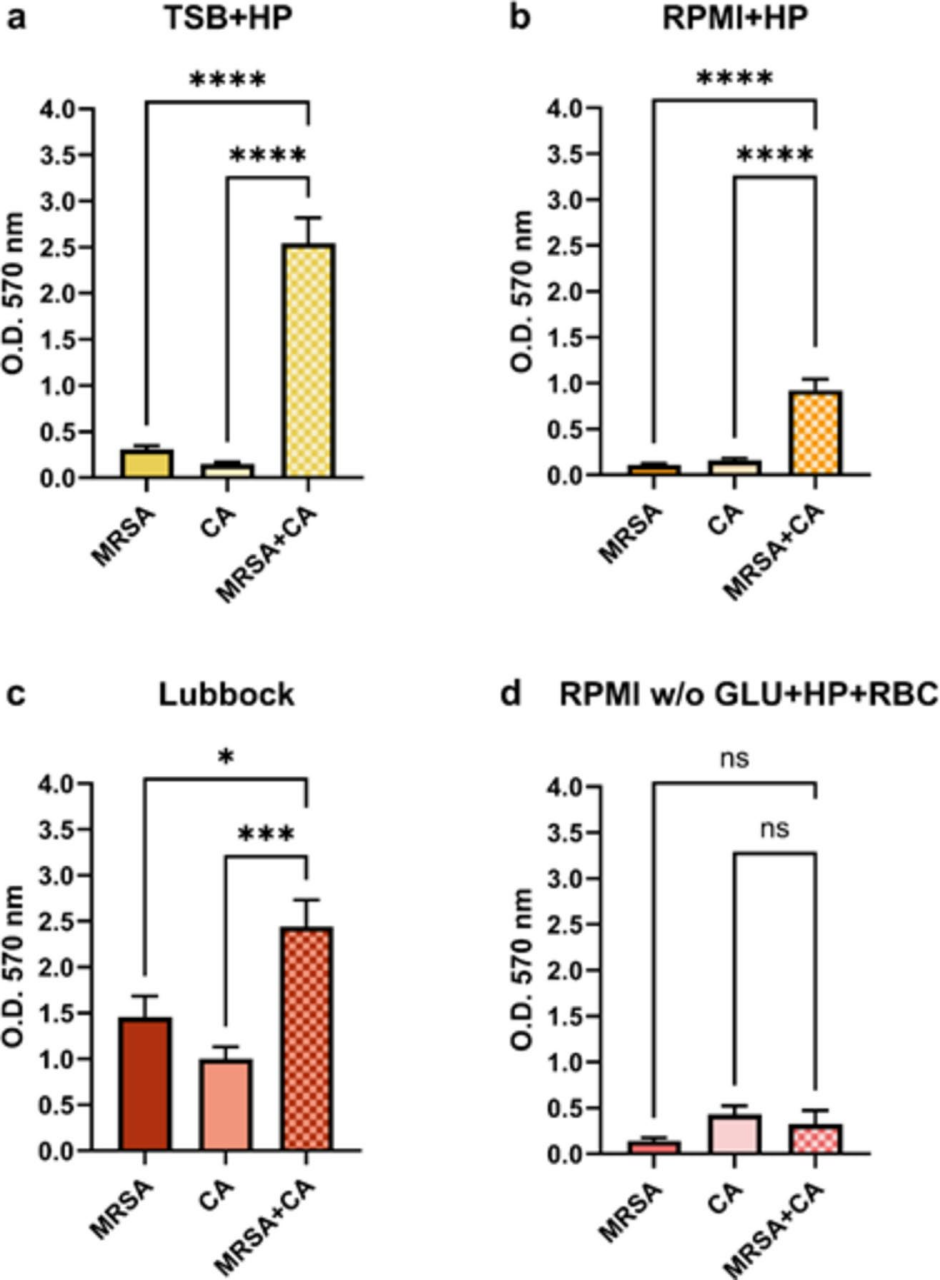
The comparison of mono-species and dual-species total biofilm biomass. The quantity of 24-hour-old total biofilm biomasses of methicillin-resistant *Staphylococcus aureus* (MRSA, ATCC 43300), *Candida albicans* (CA, ATCC 90028), and MRSA + CA dual-species biofilm consortium was evaluated with the crystal violet staining method. The values represent the mean ± SEM. Data were analyzed using one-way ANOVA, and a *p*-value < 0.05 was accepted as statistically significant. (**a**) TSB + HP – Tryptic soy broth + 10% (v/v) human plasma; (**b**) RPMI + HP – RPMI 1640 + 10% (v/v) human plasma; (**c**) Lubbock – Bolton broth + 50% (v/v) human plasma + 5% (v/v) sheep red blood cell lysate; (**d**) RPMI w/o GLU + HP + RBC – RPMI 1640 without glucose + 33% (v/v) human plasma + 5% (v/v) sheep red blood cell lysate. O.D. – optical density, ns – not significant.

As shown in Fig. 2, the cooperative interaction between MRSA and CA after a 24-hour cultivation period in three selected media (TSB + HP, RPMI + HP, and Lubbock media) seemed to be mutually beneficial. The statistically significant potentiation of dual-species total biofilm biomass production, compared to the production of total mono-species biofilm biomasses, was registered. However, after the cultivation of MRSA and CA in the last medium (RPMI w/o GLU + HP + RBC), no statistically significant difference was registered (see Fig. 2d). Evidently, the selected nutrient condition in this medium seemed to be limiting for both the mono-species and dual-species biofilm consortia formation.

### Evaluation of the impact of the four selected media on the multiplication ratios of biofilm-forming agents in MRSA + CA dual-species biofilm consortia

To calculate the multiplication ratios of biofilm-forming agents, the spread plate technique was employed to evaluate the colony-forming units (CFU) present in the initial inoculum and in dispersed, homogenized biofilm biomasses. Multiplication ratios were calculated as the ratio between the number of microbial cells present in the biofilm consortia after 24 h of cultivation and the number of cells that were present at the beginning of cultivation.

**Fig. 3:**
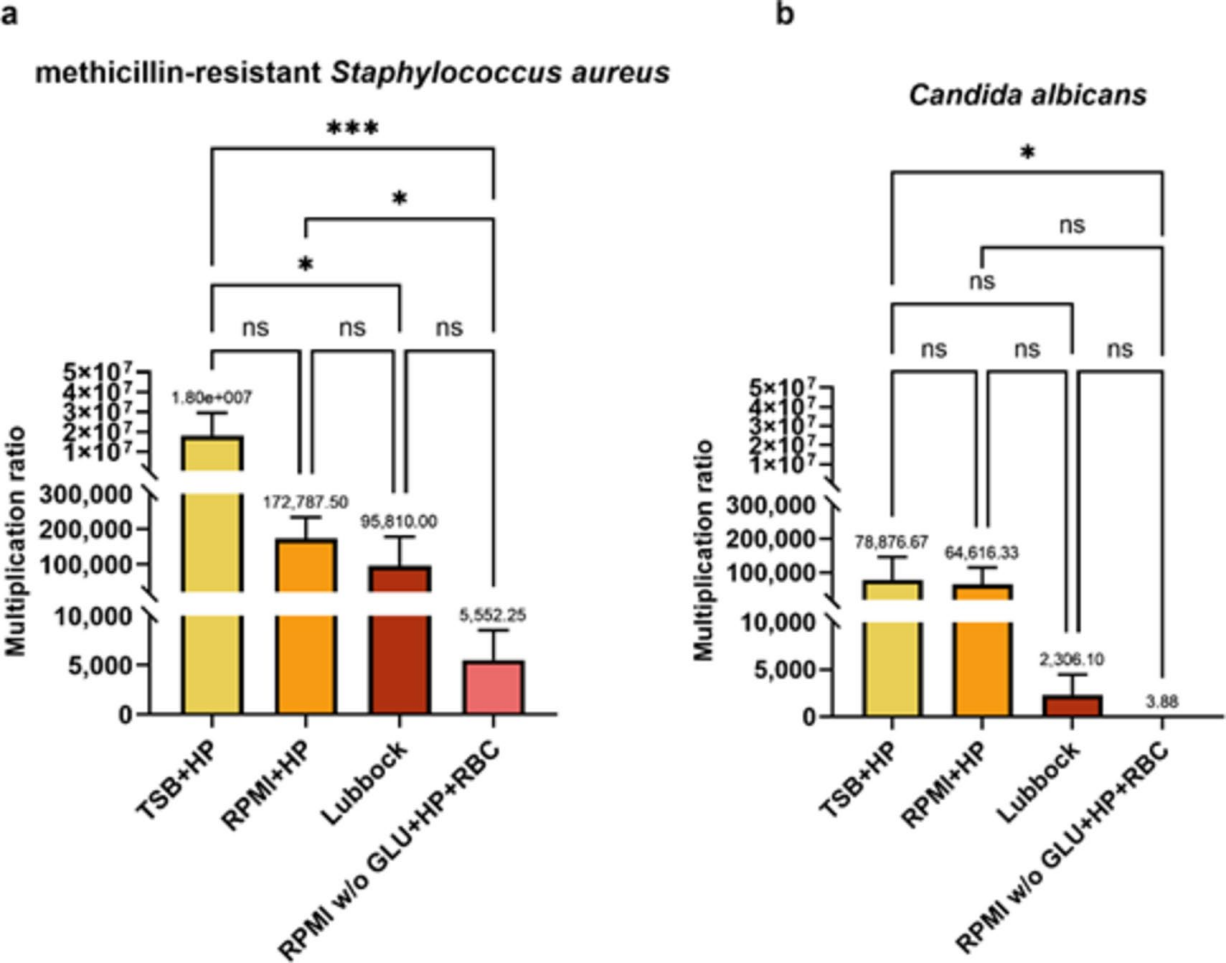
The impact of different cultivation media on multiplication ratios of individual dual-species biofilms participants. Statistical analysis corresponding to 24-hour-old dual-species biofilm consortia. (**a**) methicillin-resistant *Staphylococcus aureus* (MRSA, ATCC 43300) in dual-species (MRSA + CA) biofilm consortia; (**b**) *Candida albicans* (CA, ATCC 90028) in dual-species (MRSA + CA) biofilm consortia. The multiplication ratio represents the ratio between the number of colony-forming units/mL (CFU/mL) corresponding to individual microbial species after 24-hour cultivation and the CFU/mL of initial inoculum. The values represent the mean ± SEM. Data were analyzed using one-way ANOVA, and a *p*-value < 0.05 was accepted as statistically significant. TSB + HP – Tryptic soy broth + 10% (v/v) human plasma, RPMI + HP – RPMI 1640 + 10% (v/v) human plasma, Lubbock – Bolton broth + 50% (v/v) human plasma + 5% (v/v) sheep red blood cell lysate, RPMI w/o GLU + HP + RBC – RPMI 1640 without glucose + 33% (v/v) human plasma + 5% (v/v) sheep red blood cell lysate, ns – not significant.

As shown in Fig. 3a, the highest multiplication ratio of MRSA in MRSA + CA dual-species consortia was achieved in the TSB + HP medium. The same impact of this medium on the multiplication ratio of CA was observed (Fig. 3b). A similar impact was demonstrated for the RPMI + HP medium. No statistically significant differences compared to TSB + HP in multiplication ratios were revealed for either MRSA or CA. However, statistically significant differences were registered in the comparison of MRSA multiplication ratio in TSB + HP and Lubbock media, as well as between TSB + HP and RPMI w/o GLU + HP + RBC media. Additionally, a comparison of the RPMI + HP and RPMI w/o GLU + HP + RBC media also revealed a statistically significant difference.

When comparing multiplication ratios corresponding to CA, a statistically significant difference was registered only in a comparison of these ratios after cultivation in the TSB + HP and RPMI w/o GLU + HP + RBC media.

### Determination of the ability of MRSA + CA dual-species biofilm consortia formed in selected cultivation media to withstand exposure to selected antimicrobial drug combination

Dual-species biofilm consortia were formed for 24 h in selected cultivation media. Formed biofilms were then exposed to a combination of antibacterial and antifungal drugs ciprofloxacin (CIP) and anidulafungin (AFG). The drugs were applied at a final concentration corresponding to 50-fold of MIC (minimum inhibitory concentration of an antimicrobial drug leading to the suppression of the growth of the microbial agent in planktonic form). The MIC_CIP_ for MRSA strain corresponded to 0.256 mg/L; MIC_AFG_ for CA corresponded to 0.03 mg/L (data not shown). After 24-hour exposure, the metabolic activity of biofilm-forming cells was measured using the metabolic indicator Alamar Blue^®^.

**Fig. 4:**
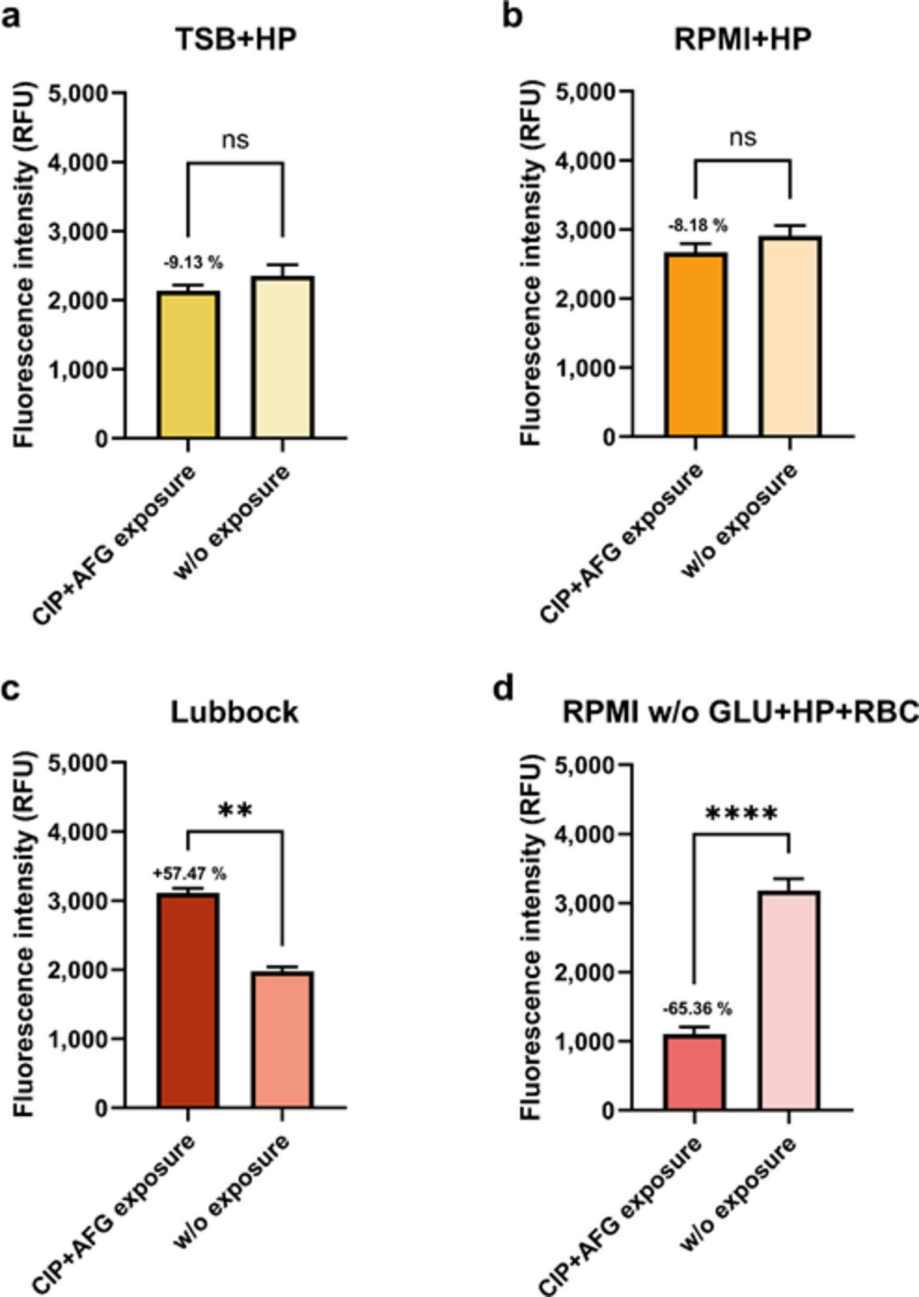
Evaluation of maintaining viability of microorganisms in dual-species biofilms. The metabolic activity of methicillin-resistant *Staphylococcus aureus* (ATCC 43300) and *Candida albicans* (ATCC 90028) in 24-hour-old biofilms before antimicrobial drugs exposure (w/o exposure) and after the next 24-hour exposure to ciprofloxacin and anidulafungin (CIP + AFG exposure) was determined using the metabolic indicator, Alamar Blue^®^. The values represent the mean ± SEM. The percentages above the columns express the difference in metabolic activity of biofilm-forming microbial participants after antimicrobial drugs exposure related to the metabolic activity of unexposed biofilm-forming microbial participants before drug exposure (100%). Data were analyzed using a t-test, and a *p*-value < 0.05 was accepted as statistically significant. (**a**) TSB + HP – Tryptic soy broth + 10% (v/v) human plasma; (**b**) RPMI + HP – RPMI 1640 + 10% (v/v) human plasma; (**c**) Lubbock – Bolton broth + 50% (v/v) human plasma + 5% (v/v) sheep red blood cell lysate; (**d**) RPMI w/o GLU + HP + RBC – RPMI 1640 without glucose + 33% (v/v) human plasma + 5% (v/v) sheep red blood cell lysate. CIP + AFG exposure corresponds to 50×MIC (minimum inhibitory concentration) of ciprofloxacin and anidulafungin. RFU – relative fluorescence units, ns – not significant.

As shown in Fig. 4a and b, the TSB + HP and RPMI + HP media support the formation of biofilms, which provided sufficient protection to their participants – no statistically significant differences in the metabolic activities of biofilm-forming agents before and after CIP + AFG drug exposure were observed. However, as shown in Fig. 4c, a statistically significant increase in metabolic activity was detected after drug exposure compared to unexposed biofilms formed in the Lubbock medium. This medium provided evident protection and enough nutrients to enable the ongoing development of the biofilm. However, the opposite situation was noted for microbial cells forming communities in the RPMI w/o GLU + HP + RBC medium (Fig. 4d). A statistically significant decrease of exposed biofilms was detected. In summary, the Lubbock medium contributed to the formation of the MRSA + CA dual-species community, which can best withstand unfavourable conditions, such as exposure to selected antimicrobial drugs (Fig. S2).

### Proving the key attributes of MRSA + CA dual-species biofilm consortia by epifluorescent microscopy

Within the epifluorescent microscopic approach, biofilm heterogeneity, the presence of a biofilm matrix, and the presence of hyphal and pseudohyphal forms of CA were evaluated. The presence of a biofilm matrix and biofilm-forming agents was examined employing a double- and triple-combination of fluorescent dyes, namely Calcofluor White (CW), SYTO 9, and propidium iodide (PI).

**Fig. 5:**
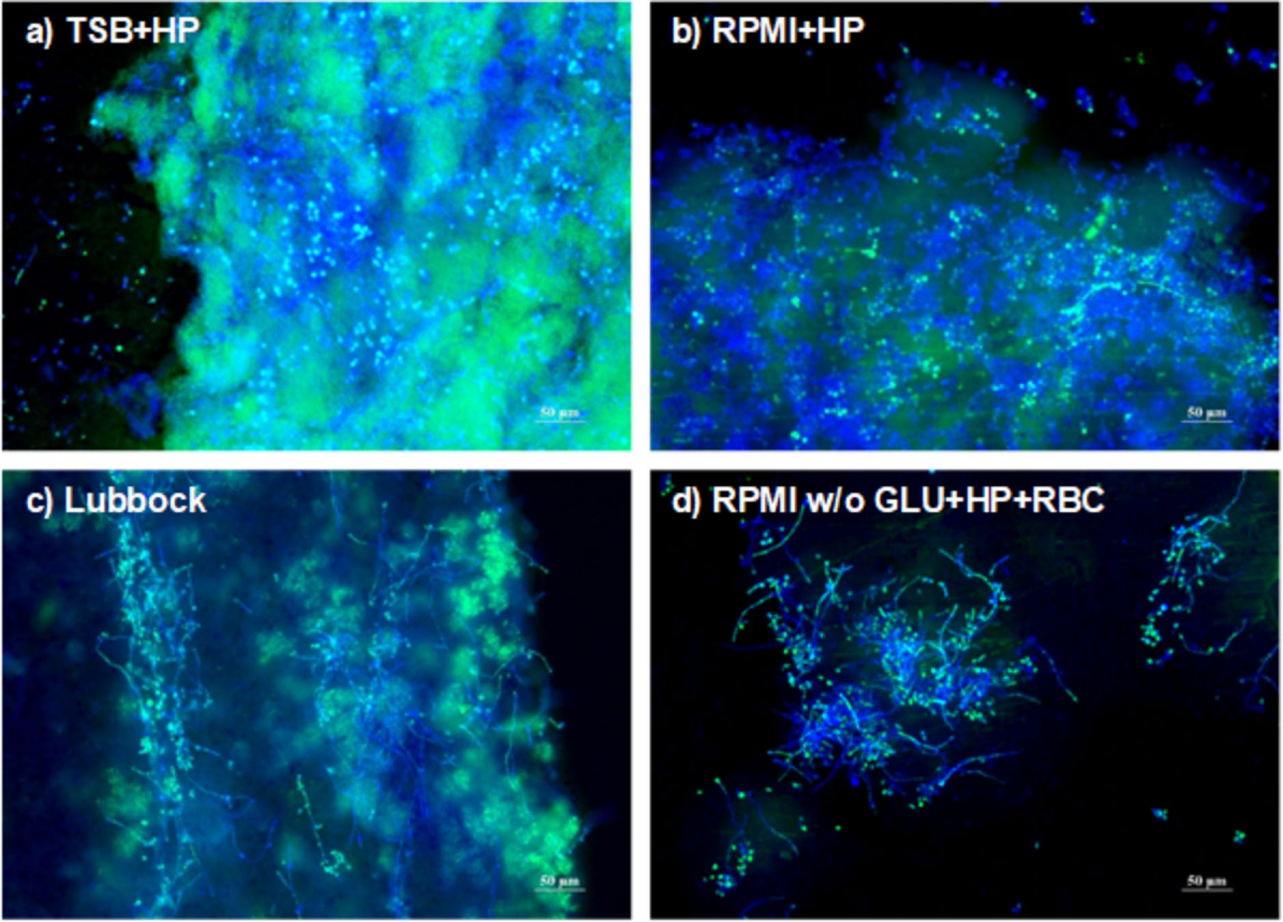
Visualization of dual-species biofilms by fluorescent microscopy (SYTO 9 + CW). Dual-species methicillin-resistant *Staphylococcus aureus* (ATCC 43300) and *Candida albicans* (ATCC 90028) biofilm consortia were formed for 24 h in the four selected cultivation media: (**a**) TSB + HP – Tryptic soy broth + 10% (v/v) human plasma; (**b**) RPMI + HP – RPMI 1640 + 10% (v/v) human plasma; (**c**) Lubbock – Bolton broth + 50% (v/v) human plasma + 5% (v/v) sheep red blood cell lysate; (**d**) RPMI w/o GLU + HP + RBC – RPMI 1640 without glucose + 33% (v/v) human plasma + 5% (v/v) sheep red blood cell lysate. Double-combination of fluorescent dyes, Calcofluor White and SYTO 9 were employed for microbial cells (blue yeast cells, green bacterial cells) and biofilm matrix (blue, green stained unbounded, dispersed mass) visualization. The scale bar corresponds to 50 μm.

A greater content of biofilm matrix (dense, dispersed mass, without any clear boundaries) was evident in the biofilms formed in the TSB+HP and the Lubbock media (Fig. 5a and c, and Supplementary information Figs. S3a, S3c). These findings fully correlated to the inspection of the formed biofilm biomasses with the naked eye (see Fig. S1, biofilm matrix is responsible for the slime-like texture of the consortia). A lower density of biofilm matrix was observed in the communities formed in the RPMI+HP medium (Fig. 5b and Supplementary information, Fig. S3b). In the biofilm consortia formed in RPMI w/o GLU+HP+RBC, a mass corresponding to the biofilm matrix was present only rarely (Fig. 5d and Supplementary information, Fig. S3d). The transition of the yeast CA to a pseudohyphal or hyphal form was observed mostly in the consortia formed in the Lubbock medium and the RPMI w/o GLU+HP+RBC medium.

### Quantification of dominant matrix biomolecules of MRSA + CA dual-species biofilms

After biofilm homogenization and matrix extraction, the key components of the biofilm matrix, namely carbohydrates, proteins, and eDNA were quantified using commercially available kits. Considering that the biofilm consortia formed in RPMI w/o GLU + HP + RBC did not achieve the desired attributes (a non-biofilm producer phenotype of MRSA + CA dual-species biofilm consortia, the presence of a matrix in low amounts), the biofilm matrix of these communities was excluded from further analysis.

**Fig. 6:**
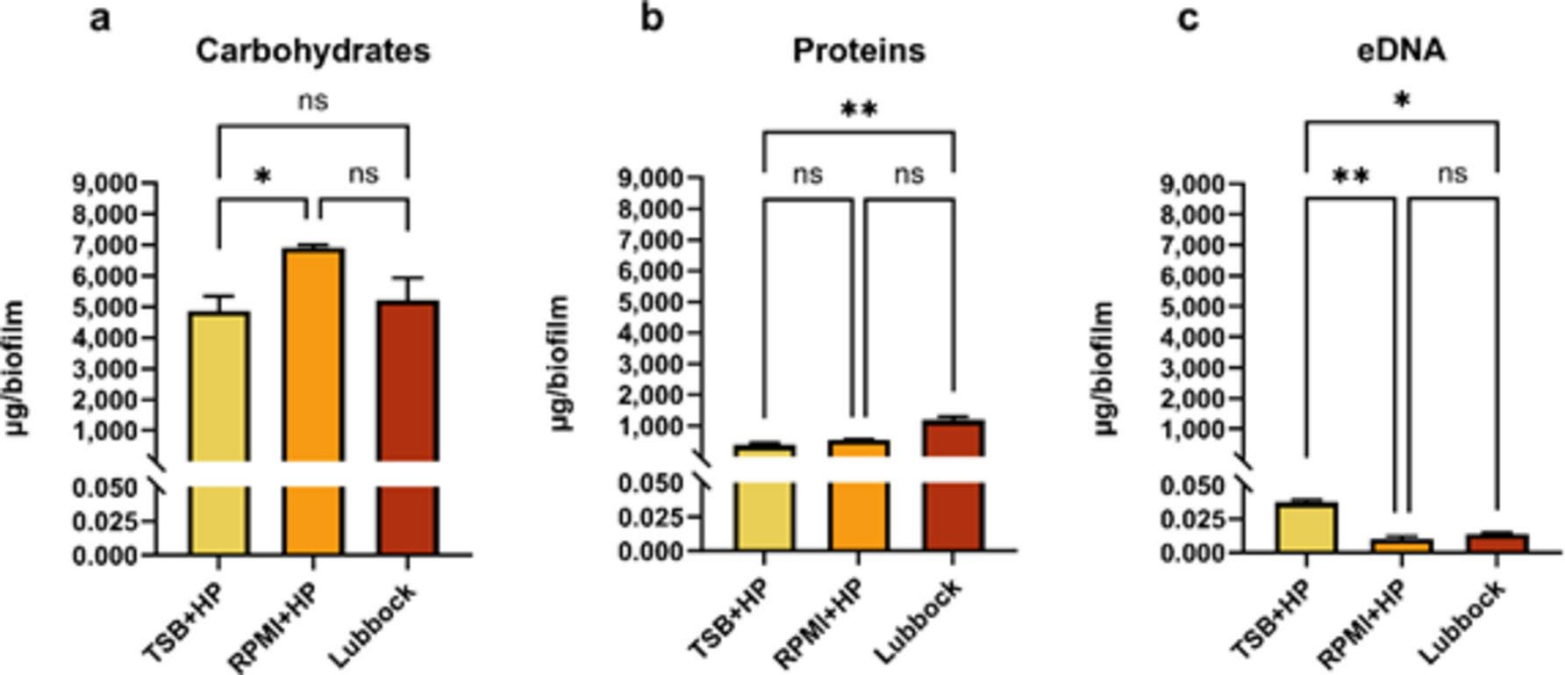
Comparative analysis of concentrations of carbohydrates, proteins, and eDNA in dual-species biofilms. Dual-species, methicillin-resistant *Staphylococcus aureus* (ATCC 43300) and *Candida albicans* (ATCC 90028), biofilm matrices were analysed after 24 h of biofilm formation in three selected cultivation media: TSB + HP – Tryptic soy broth + 10% (v/v) human plasma; RPMI + HP – RPMI 1640 + 10% (v/v) human plasma; Lubbock – Bolton broth + 50% (v/v) human plasma + 5% (v/v) sheep red blood cell lysate. The values represent the mean ± SEM. Data were analysed using one-way ANOVA and a *p*-value < 0.05 was accepted as statistically significant. eDNA – extracellular DNA, ns – not significant.

In all evaluated biofilm matrices, carbohydrates were among the dominant biomolecules (see Supplementary information, Fig. S4). As shown in Fig. 6, a comparative analysis of carbohydrate concentrations revealed a statistically significant lower concentration only in the matrices of the biofilms formed in the TSB + HP medium compared to the matrices formed in the RPMI + HP medium. The greatest presence of proteins in biofilm matrices was recognised in the biofilm consortia formed in the Lubbock medium. A comparative analysis of protein concentrations revealed a statistically significant difference only in the biofilm matrices formed in the Lubbock and TSB + HP media. The highest statistically significant concentration of eDNA was registered in the biofilm matrices formed in the TSB + HP medium.

## Discussion

Microbial biofilm-associated infections represent a significant clinical challenge and a legitimate reason for focusing the attention of the research society^25^. One of the current pitfalls in anti-biofilm research is the low correlation of the results from the screening of in vitro anti-biofilm efficacy and the results from advanced preclinical, or clinical trials^19^. It has been demonstrated that microbial biofilms are often comprised of different microbial species^7^. An example of these mixed biofilms can be communities represented by inter-kingdom MRSA and CA partners, participating in difficult-to-treat, mixed infections of wounds^17^. Environmental conditions, including nutrient availability, are reflected in the entire biofilm community. These conditions influence the production of a biofilm matrix, which plays a crucial role in the adaptive resistance of biofilm communities^26,27^.

The primary aim of this study was to establish nutritional conditions that would lead to the production of an inter-kingdom MRSA + CA biofilm consortia in vitro. This biofilm should possess a high degree of tolerance to antimicrobial drugs with an evident contribution of the biofilm matrix. In addition, the impact of an effort to induce glucose and nitrogen nutrient availability corresponding to human wound exudate on MRSA + CA biofilm formation in vitro was assessed.

MRSA + CA dual-species biofilms were formed in four nutritionally different cultivation media in vitro. The formed MRSA + CA biofilms were compared by evaluating several key attributes. These included the multiplication ratios of participating microorganisms, the quantitative representation of key biomolecules in the biofilm matrix, and the ability to withstand exposure to selected antimicrobial drugs.

The compositions of cultivation media were as follows: The first medium was TSB supplemented with 10% (v/v) HP. The TSB medium represents a general-purpose, nutritious medium that supports the growth of both non-fastidious bacteria and yeasts^23^. For SA biofilm studies, supplements such as glucose, haemoglobin, or human plasma are added to the medium based on TSB^22,28–30^. Similarly, TSB represents the most used medium for *Candida* spp. and mixed inter-kingdom biofilm studies^23,31^.

The second medium chosen was based upon RPMI, supplemented with 10% (v/v) HP. The RPMI medium also belongs to a wide range of culture media chosen by researchers not only for *Candida* spp. but also for mixed bacterial-fungal biofilm formation and biofilm studies in vitro^29,31,32^.

Next, the Lubbock medium consisted of a Bolton broth medium, 50% (v/v) HP, and 5% (v/v) RBC. This medium is used as a versatile in vitro wound-simulating medium in Lubbock chronic wound biofilm models^21,33^.

The last medium consisted of the RPMI broth w/o GLU. To reproduce a chronic human-like wound environment, namely wound exudate composition, the RPMI broth was supplemented with 33% (v/v) HP, and 5% (v/v) RBC^34–39^.

All employed media were supplemented with HP. It was registered that in staphylococci HP induces a significant increase in the expression of genes encoding adhesive molecules, MSCRAMMs (microbial surface components recognising adhesive matrix molecules). These molecules participate in the early stages of biofilm formation by mediating the attachment of staphylococci to the host structures. In addition, it was also demonstrated that staphylococcal biofilms produced in the presence of HP showed significantly reduced susceptibility to antibacterial drugs^40^. The impact of HP on the yeast CA was also demonstrated. For instance, it was revealed that HP induces a yeast-to-hyphae transition in CA. Surface coating with HP also contributes to a significant adhesion of germinated yeast cells^41^. Although there are some discrepancies concerning the contribution of the blastospore vs. hyphal form of CA in biofilm formation, the crucial contribution of the hyphal form in biofilm formation was recognised in all studies^42,43^.

Different host-derived immune effector molecules are present in HP. These molecules include immunomodulating proteins such as antibodies, complement proteins, antimicrobial peptides, and cytokines^44^. Dynamic complex interplay occurs between these effector molecules and biofilm-forming microorganisms within the host environment. Ultimately, the impact of host effector molecules can either promote or inhibit microbial biofilm formation. A detailed understanding which host molecules influence biofilm formation is crucial for the research and development of alternative anti-biofilm strategies aimed at modulation of the immune response. The general goal of these alternative treatment strategies is to block the host molecules promoting microbial biofilm formation and subsequently reduce the formation of persistent biofilms. In conclusion, understanding how host effector molecules interact with biofilm-forming microorganisms and microbial biofilms, can provide valuable insight for the alternative management of biofilm-associated infections^45,46^.

Two selected media, namely the Lubbock and RPMI w/o GLU + HP + RBC were additionally supplemented with RBC. Haemoglobin released from disrupted red blood cells represents the most abundant iron source in hosts. In some infection scenarios, e.g. wound infections, bacteria are exposed to abundant amounts of host hem-iron molecules. These circumstances can significantly contribute to the regulation of virulence factors and the promotion of microbial pathogen adherence^47^.

Different interactions such as synergy, mutualism, syntropy, and antagonism can occur in mixed-species biofilm consortia. These interactions are complex and involve metabolic cooperation, community-coordinated gene expression, and competition for space and nutrients^8^.

A significant increase of MRSA + CA dual-species total biofilm biomasses, compared to the production of MRSA or CA mono-species biofilm biomasses, was registered after the cultivation of MRSA and CA in the TSB + HP, RPMI + HP, and Lubbock media. Therefore, we suggest that these media might provide favourable nutritional conditions for potential synergistic interactions between microbial biofilm participants, MRSA and CA. On the contrary, an apparently unfavourable mutual interaction between MRSA and CA was registered after the cultivation of these agents in the RPMI w/o GLU + HP + RBC medium. Nevertheless, the greatest total biofilm biomasses were registered after the cultivation of MRSA + CA in the TSB + HP and Lubbock media.

However, the beneficial interaction of microbial partners in biofilm communities cannot be deduced only from the robustness of biofilm biomasses. It is necessary to perceive the benefit of ”living together“ in microbial communities as the ability to resist hostile conditions. The biofilm matrix contributes to this ability extraordinary^5^.

It has been suggested that the biofilm matrix plays a ”glue-like“ function and contributes to the heterogenous, layered arrangement of the whole consortium. Biofilm participants in upper biofilm layers have more favourable conditions for sustaining life than participants present in deeper layers, where hostile conditions prevail with a limited availability of oxygen and nutrients. However, microbes can effectively adapt to these adverse conditions by the transition to persistent states^4^. Persistent cells are metabolically less active subsets of phenotypic variants, highly tolerant to antimicrobial drugs. They are also recognised as a major contributor to the chronic stage of biofilm-associated infections. The biofilm matrix acts as a diffusion-limiting barrier that restricts antimicrobial drug penetration^2^. Furthermore, antimicrobial drugs that can pass through this barrier into the deeper layers of the biofilm consortium can be degraded by secreted microbial enzymes which are accumulated in the biofilm matrix^4^.

As shown in the results, it is evident that the highest amount of protection without any limitation of ongoing progress or the development of the biofilm community was achieved after antimicrobial drug exposure in the consortia formed in the Lubbock medium. Sufficient protection against unfavourable conditions was also achieved in the biofilm communities formed under the nutrition conditions provided by the TSB + HP and RPMI + HP media, where viability was maintained. On the contrary, significantly reduced metabolic activity was registered in the consortia formed in the RPMI w/o GLU + HP + RBC medium.

The most favourable conditions for the multiplication of both MRSA and CA in the dual-species biofilm consortia were apparent in the TSB + HP medium. Therefore, it can be concluded that comparing the selected media in this study, only this medium is the best nutritionally balanced (nitrogen sources and glucose) for the multiplication of both employed biofilm-forming agents. On the contrary, the RPMI w/o GLU + HP + RBC medium appears to be nutritionally insufficient for MRSA + CA dual-species biofilm formation. Apparently, glucose availability plays a significant role here. Generally, for most pathogens, glucose represents the main source of energy and a fundamental building block for survival and multiplication^48^. It was also demonstrated that glucose was an effective supplement for enhancing staphylococcal biofilm biomass formation in vitro and was also required for *Candida* spp. biofilm formation^49,50^.

Within the evaluation of the individual microorganisms CFU in the formed biofilm consortia and the consequent evaluation of the multiplication ratios, evident differences were registered in the TSB + HP and Lubbock media. However, based on the quantification of the total biofilm biomasses (biofilm-forming agents and the biofilm matrix together) the “strong biofilm producers“ categorization was recognised for microbes cultivated in both media. According to the above-mentioned findings, it can be hypothesized that the tolerance of microbial consortia formed in the Lubbock medium to anti-infective drugs was not only associated with total microbial load but also the presence of the biofilm matrix played a key role in the ability to withstand induced hostile conditions.

In the microbial biofilm matrices, the major EPS include biomolecules such as polysaccharides, proteins, and eDNA^4^. Therefore, for biofilm matrix visualization, fluorescent dyes binding to matrix polymers should be employed. CW binds to polysaccharide polymers (typically present in biofilm matrices and fungal cell walls), and is commonly used for the visualization of mycotic agents^51^. Biofilm-forming bacterial cells were stained by SYTO 9 and PI. These fluorescent probes bind to nucleic acids present in microbial cells. However, it should be noted that these nucleic acids are also present in extracellular environments (eDNA is a biofilm matrix biomolecule)^52^. By evaluating MRSA + CA biofilms with epifluorescence microscopy, the highest density of matrix and the greatest biomass heterogeneity were observed in the consortia formed in the TSB + HP and the Lubbock media. Within this investigation, attention to the morphology of the yeast CA was also paid. Two main morphological types of CA can be recognised. Blastospore is a form with dissemination potential, and the hyphal or pseudohyphal form is considered a virulence factor and a risk factor for the development of life-threatening invasive candidiasis, e.g. in wound biofilm-associated infections^53^. The virulent, pseudohyphal/hyphal form of CA was observed, especially in the biofilm consortia formed in the Lubbock and RPMI w/o GLU + HP + RBC media.

The complexity of polymicrobial biofilm-associated infections and the cumulative presence of embedded pathogens in infectious biofilm deposits present significant challenges. Additionally, the protective shield formed by these consortia in the biofilm matrix adds to the difficulty. Together with the AMR of the pathogens themselves, this creates an even greater challenge in finding an efficient treatment strategy. Therefore, for effective eradication of infectious biofilm deposits, it is necessary to target both the structurally complex extracellular matrix and the pathogens themselves. For potentiating biofilm antimicrobial eradication, strategies of EPS weakening and degradation are the logical solution to this clinical therapeutic challenge^54^. Until now, some perspective therapeutic strategies targeting EPS have been reviewed. However, the knowledge acquired has been derived from monospecies biofilms and studies focused on poly-species systems remain sparse^4^.

The biofilm matrix generally consists of macromolecules, such as exopolysaccharides, proteins, and nucleic acids^4^. It was revealed that the structural and biochemical properties of the biofilm matrix, as well as the quantitative profile of its macromolecules, differ significantly due to various biological and environmental factors. The differences in matrix composition are mainly driven by the genetic diversity of the microbial participants in biofilm consortia. This diversity includes microorganisms from different biological kingdoms, species, and even genetic strains. Other biological factors influencing the matrix composition include the presence of other microorganisms in biofilm consortia or surrounding environment, as well as host immune defense mechanisms. Key environmental factors include nutrient availability, temperature, pH, or oxygen availability^17,55^.

Quantification procedures using available commercial kits were performed in this study to reveal which basic matrix molecules should be targeted in MRSA + CA biofilm communities. It was shown that the most abundant biomolecules in all inspected biofilm matrices formed in the TSB + HP, RPMI + HP, and Lubbock media were carbohydrates. The highest concentration of proteins was recognised in the matrix formed in the Lubbock medium. This result is not that surprising. The HP suplement in the Lubbock medium had the highest content (50% v/v) compared to the other employed media. SA can gain a significant advantage over the host defence mechanisms by manipulating the coagulation system of the host. SA is a coagulase-positive bacterium, capable of producing enzymes (e.g. coagulase) that stimulate the clot formation of plasma components^56^. Generally, host proteins and host cell debris were found to contribute to the biofilm matrix scaffold as well^33,56^. As it was evident from a visual inspection of the formed biofilm biomass, supplements present in the Lubbock medium, namely HP and RBC, were utilized to create a protective barrier for this MRSA + CA consortium. In contrast, the RPMI w/o GLU + HP + RBC medium did not activate the MRSA ability to convert the available human plasma proteins into protective clot formation, although the proteins were also present at a higher concentration (33% v/v of HP). It could be hypothesized that the limiting nutrition availability of a primary source of energy for the metabolic processes of microbial cells, glucose, might play a role in this inability.

Indeed, we can summarize that the medium with the most similarity to nitrogen and glucose availability to human wound exudate (RPMI w/o GLU + HP + RBC) did not support the formation of an inter-kingdom complex biofilm community of MRSA and CA in vitro. We might propose that this result was associated with the low availability of glucose in the medium. From the selected media included in this study, the Lubbock medium provided the most appropriate nutrition conditions for forming MRSA + CA dual-species biofilms in vitro. The MRSA + CA biofilm consortia formed in this medium showed the highest degree of tolerance to selected antimicrobial drugs with the evident contribution of the biofilm matrix. The three-dimensional biofilm biomass structure responsible for adaptive resistance mechanisms was already evident to the naked eye. In addition, an evaluation by fluorescent microscopy showed the apparent heterogeneity of the biofilm biomass, a high-density matrix, and the presence of morphological form of CA corresponding to the virulent state.

In conclusion, the Lubbock medium was recognised as the most appropriate medium for the formation of MRSA + CA dual-species consortia *in vitro.* Interestingly, regardless of the nutrition conditions, the dominant matrix biomolecules in the MRSA + CA inter-kingdom communities were carbohydrates. These findings could be considered as one of the additional antibiofilm therapy targets. Surprisingly, the medium with the most similarity to nitrogen and glucose availability to human wound exudate was inappropriate for in vitro MRSA + CA dual-species biofilm formation. Thus, the results from this study contribute to the valid and rational employment of nutrition conditions supplemented with host effector molecules within MRSA + CA dual-species biofilm formation in vitro. This is important for subsequent preclinical research of anti-biofilm strategies.

## Methods

### Microbial strains and cultivation media

Two reference microbial strains, the bacterium MRSA (ATCC 43300, CCM 4750), and the yeast CA (ATCC 90028, CCM 8261), purchased from the Czech Collection of Microorganisms (CCM, Czech Republic), were employed in this study. To avoid genetic and epigenetic changes, which can occur due to multiple passages under in vitro conditions, we performed all experiments only with cryopreserved stock microbial cultures.

Four cultivation media were included in the study (for more details, see Supplementary information, Table S1), namely TSB (Himedia, India), supplemented with 10% (v/v) HP (Biowest, France), pH 7.3±0.2; a chemically defined RPMI medium with L-glutamine, sodium bicarbonate, and glucose (2 g/L) (Merck, USA), supplemented with 10% (v/v) HP, pH 7.4±0.2; the Lubbock medium (sheep red blood cells were used instead of horse red blood cells) consisted of Bolton broth (Merck, USA), supplemented with 50% (v/v) HP, and 5% (v/v) RBC (LabMediaServis, Czech Republic), pH 7.4±0.2^21^; and a chemically defined RPMI medium with L-glutamine, sodium bicarbonate, w/o GLU (Biowest, France), supplemented with 33% (v/v) HP, and 5% (v/v) RBC, pH 7.2±0.2.

### Culture microtiter plate method for MRSA and CA mono- and MRSA + CA dual-species biofilm production in vitro

Before each experiment, cryopreserved stocks of bacterial/yeast suspensions were inoculated on Mueller Hinton agar/Sabouraud dextrose agar (Himedia, India) and incubated at 37 °C in a humid atmosphere for 16–24 h. After that, the homogenous suspensions of bacteria/yeasts with optical density (O.D., 565±15 nm, DEN-1, BioSan, Latvia) equal to O.D. = 0.5 McFarland units were prepared in individual cultivation media. The microdilution method and spread plate technique for evaluating CFU in initial inocula were employed. The number of MRSA cells in homogenous suspensions (O.D. = 0.5 McFarland units) corresponded to ≈1.15 × 10^7^ CFU/mL. In the yeast homogenous suspensions (O.D. = 0.5 McFarland units), the number of cells corresponded to ≈1.07 × 10^6^ CFU/mL.

Before all experiments related to mono- and dual-species biofilm formation, the microtiter plate well surfaces were modified by HP preconditioning. For plastic surface coating, all wells were treated with 200 µl of HP for 24 h at 37 °C. After the coating process, HP was removed, and the wells were left to air dry.

For MRSA and CA mono-species biofilm formation, individual wells were filled with 200 µl of homogenous suspensions in appropriate cultivation media. For dual-species biofilm formation, individual wells were filled with 200 µl of homogenous suspensions at a volume ratio of 1:1 (100 µl of bacterial suspension and 100 µl of yeast suspension). After that, the plates were incubated for 24 h on a rocking Table (25 rpm, Mini Rocker Shaker MR-1, BioSan, Latvia) at 37 °C in a humid atmosphere.

### Assessment of biofilm biomass formation by the CV staining method

The quantitative test described by Christensen *et al.*^56^ was employed for quantification of the total biofilm biomasses and subsequent biofilm producer phenotype categorization. After a cultivation period of 24 h, the formed biofilms were rinsed three times using a sterile 0.9% saline solution and then left to air dry. Subsequently, adherent biofilms were fixed with ice-cold methanol for 15 min at 4 °C. Then, an aliquot of 200 µl of the 0.05% CV solution per well was added and incubated at room temperature for 30 min on a rocking table. Excess stain was removed, and the wells were gently rinsed three times with deionized sterile water. The fixed CV stain was eluted by an ethanol/acetone mixture (80:20) at room temperature for 30 min on a rocking table. The O.D. of the dissolved CV was read at 570 nm using a microplate reader (Synergy HTX Multimode reader, BioTek, USA).

### Recognition of the biofilm producer phenotype for mono- and dual-species biofilm communities

The biofilm-forming microbes forming mono- and dual-species biofilm consortia were categorized into biofilm producer phenotype according to the measured O.D. of eluted CV (O.D.cv) that was bound to the produced biofilm biomasses.

The same criteria for biofilm phenotype categorization, as described previously by Stepanović *et al.*^24^ was employed. Namely, four categories were introduced: if O.D.cv ≤ O.D.c (O.D. of the negative control), the strains were classified as non-biofilm producers (-). If O.D.c < O.D.cv ≤ 2×O.D.c, the strains were considered weak biofilm producers (+); 2×O.D.c < O.D.cv ≤ 4×O.D.c were included in the group of moderate biofilm producers (++) and if the condition 4×O.D.c < O.D.cv was met, the strains were considered strong biofilm producers (+++).

### Determination of the metabolic activity of MRSA + CA dual-species biofilm participants after exposure to selected antimicrobial drugs combination

Two commercially available antimicrobial drugs, the antibiotic drug CIP and the antimycotic drug AFG, both from Merck, USA, were employed to determine the ability of the biofilm consortia to withstand hostile conditions created by antimicrobial drug exposure. At first, the microdilution broth method according to EUCAST^57,58^ was employed for the determination of the MIC to the planktonic form of MRSA (MIC_CIP_) and CA (MIC_AFG_). Dimethyl sulfoxide was used as a cosolvent of CIP and AFG (the final concentration did not exceed 1%).

The next procedure was as follows: the MRSA + CA dual-species biofilm consortia were formed in selected media as described above (see chapter Culture microtiter plate method for MRSA and CA mono- and MRSA + CA dual-species biofilm production in vitro). The 24-hour-old biofilm biomasses were rinsed three times with a sterile 0.9% saline solution. After that, the antimicrobial drugs, CIP in Cation-adjusted Mueller-Hinton broth (CAMHB, M-H 2 Broth, Merck, United States), and AFG in the RPMI medium supplemented with L-glutamine, sodium bicarbonate, and glucose (2 g/L) were added at a volume ratio of 1:1 (the final volume corresponded to 200 µl) into each well. The final concentration of CIP and AFG corresponded to 50×MIC of individual drugs (CIP = 12.8 mg/L, AFG = 1.5 mg/L).

After 24-hour exposure (cultivation of the exposed biofilm-forming microbes in a humidified atmosphere at 37 °C), the media containing antimicrobials were replaced by a fresh TSB medium. Two short sonication steps were included (each for 5 min). For another restoration of metabolic activity (especially in the persistent cells), incubation for 75 min in a humidified atmosphere at 37 °C was performed. Subsequently, 20 µl of the working solution of metabolic indicator, Alamar Blue^Ⓡ^ (a 1:1 mixture of 0.02% Alamar Blue^Ⓡ^ Cell Viability reagent, Thermo Fisher Scientific, USA, and 10% Tween 80), was added into each well. The plates were incubated for 30 min at 37 °C in a gentle shaking mode. After the incubation period, a fluorescence measurement (λ_Ex_ 530 nm and λ_Em_ 590 nm, microplate reader Synergy HTX Multimode reader, BioTek, USA) was taken. The metabolic activity of the microbial biofilm participants before drug exposure (24-hour-old biofilms without any drug exposure/unexposed control) was processed analogously. Briefly, after 24 h of biofilm formation in the selected media, all media were removed and replaced by fresh TSB. Subsequently, the biofilm consortia were disrupted by sonication steps, the metabolic indicator was added, and after incubation, the fluorescence intensity was measured.

### Extraction and quantification of principal matrix biomolecules and an evaluation of the multiplication ratios of MRSA and CA in MRSA + CA dual-species biofilms

For extraction and further quantification of the key components of the biofilm matrices of MRSA + CA dual-species biofilms, biofilm formation was performed in 24-well sterile, flat-bottom polystyrene plates (Merck, USA). Briefly, the wells were filled with bacterial and yeast suspensions in appropriate cultivation media at a ratio of 1:1 (0.9 mL of bacterial suspension + 0.9 mL of yeast suspension, both with O.D. = 0.5 McFarland units). The same cultivation conditions, including the plastic surface coating (as mentioned in chapter Culture microtiter plate method for MRSA and CA mono- and MRSA + CA dual-species biofilm production in vitro) were established for biofilm formation. After 24 h cultivation, the formed biofilms were rinsed three times with a sterile 0.9% saline solution and left to air dry. 0.5 mL of sterile 0.9% saline solution was added into each well, and then the biofilms were properly scraped, transferred into a falcon tube (VWR, USA), and homogenized for 1 min by a Handheld Homogenizer (MT-30 K, Miulab, China) at 18 000 rpm.

For the disintegration of the carbohydrate and protein moiety of the biofilm matrices, and biofilm-forming cell dispersion, α-amylase from *Aspergillus oryzae* and proteinase K from *Tritirachium album* (both Merck, USA) were employed. At first, the amylase at a final concentration of 20 mg/mL in 20 mM Tris-HCl buffer with 100 mM NaCl (pH 7.4) was added to mechanically disintegrated biofilm biomasses and incubated for 45 min at 37 °C. Next, the proteinase at a final concentration of 200 µg/mL in 20 mM Tris-HCl buffer with 3 mM CaCl_2_ (pH 7.4) was added, and the incubation step followed for the next 45 min at 37 °C. Afterward, two sonication steps (each for 5 min) were performed. Finally, the disintegrated biofilm biomasses were centrifuged (Hettich, Germany) at 10 000 × g for 15 min (24 °C), and the supernatants were transferred to test tubes as extracellular matrix fractions (ECM). The ECM were subsequently filter-sterilized (0.22 μm pore size). The pellets were further processed using the microdilution method and spread plate technique for an evaluation of MRSA and CA CFU.

Commercially available kits were employed to quantify the key EPS compounds in ECM. For carbohydrates: Total Carbohydrate Assay Kit (Merck, USA), proteins: Pierce™ BCA Protein Assay Kit (Thermo Fisher, USA); and for eDNA: Quant-iT™ PicoGreen™ dsDNA Reagent and Kit (Thermo Fisher, USA) were used. All analyses were performed according to the manufacturers’ instructions.

Pelleted bacteria and yeasts were resuspended in a sterile 0.9% saline solution and subjected to serial dilution. The diluted suspensions of the microorganisms were plated on Mueller Hinton agar (MRSA) or Sabouraud dextrose agar (CA). The CFU were counted after cultivation for 24 h at 37 °C.

### Visualization of the MRSA + CA dual-species biofilm matrix and biofilm-forming microorganisms by epifluorescent microscopy

Visualization of the biofilm matrices and biofilm-forming microorganisms was performed using SYTO 9 fluorescent stain (a final concentration of 4.90 µM). This stain was used to detect the DNA of living cells and eDNA in the biofilm matrix. PI (a final concentration of 19.59 µg/mL) was used to detect the DNA of dead cells and eDNA in the biofilm matrix (both Invitrogen, USA). CW was used to detect yeasts and polysaccharides in the biofilm matrix (Merck, USA).

At first, 24-hour-old biofilms were rinsed three times with a sterile 0.9% saline solution. The mixture of SYTO 9 and PI was added to each well to completely cover the biofilms, followed by a 30-minute incubation in the dark with gentle shaking at room temperature. After the staining procedure, the biofilms were scraped off and transferred to a microscopic slide. In the case of combination with CW, two drops of CW were added. Subsequently, the specimen was covered with a cover glass. The stained biofilms were imaged by epifluorescence microscopy with an Olympus Provis fluorescent microscope (Olympus, Japan), equipped with a photographic device (DS-Fi3, Nikon, Japan). The images were collected and adjusted using NIS-Elements software, version 5.00 (Laboratory Imaging, Czech Republic).

### Statistical analysis

For the study, measured data collected from three independent experiments were included in the statistical analysis. For the crystal violet staining method, eight replicates, and for all other experiments, six replicates were involved in each experiment. O.D. or RFU (relative fluorescent units) values corresponding to negative controls (individual cultivation media) were subtracted from all experimental data. The statistical analysis was performed using GraphPadPrism software version 9 (GraphPad Software, Inc., USA). One Way Analysis of Variance (ANOVA), or t-test were performed to evaluate significant differences. A *p*-value < 0.05 was accepted as statistically significant. Data are presented as mean ± SEM.

**Fig. 7:**
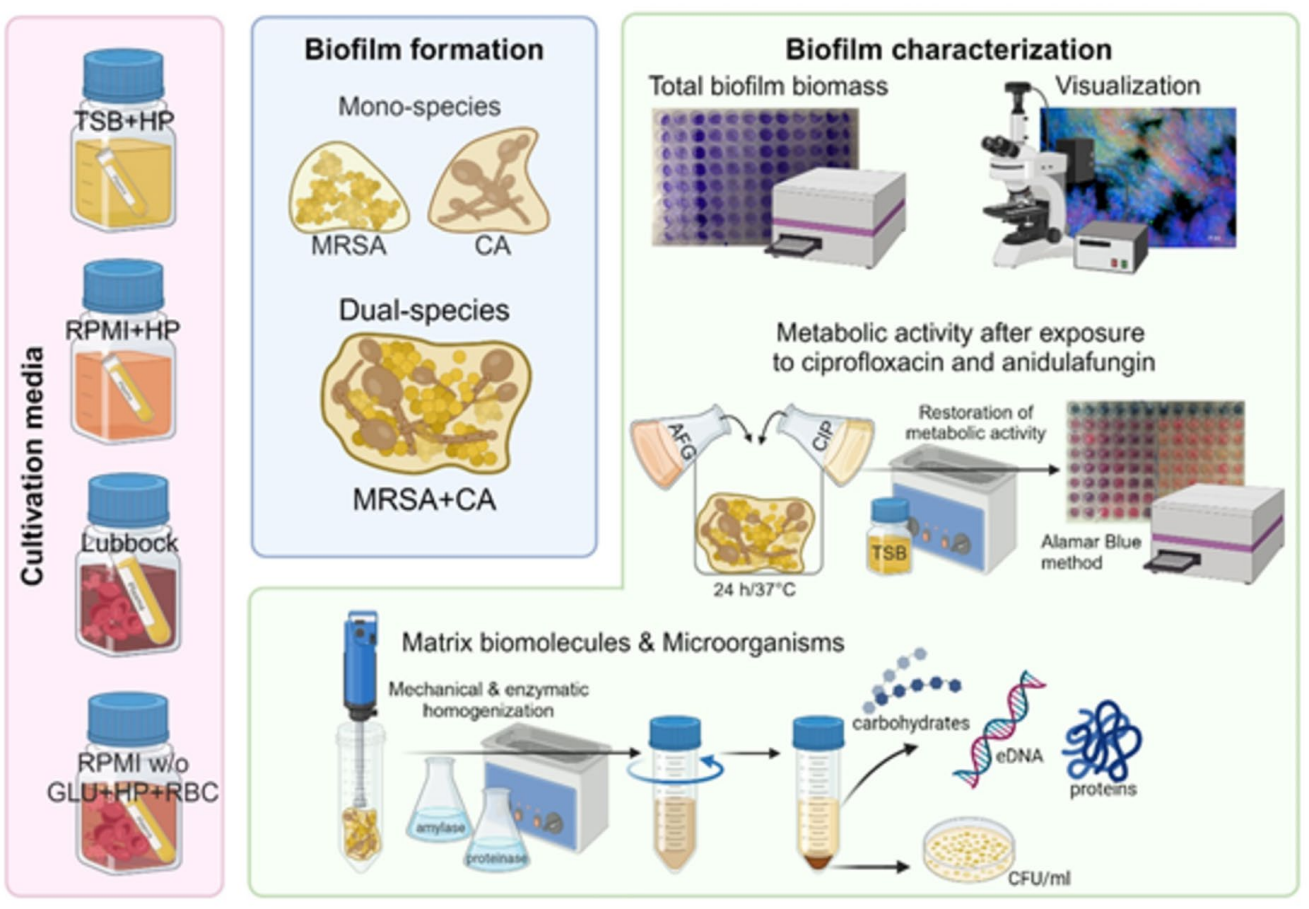
Flowchart of methods used for formation and comparison of mono- and dual-species biofilms. TSB + HP – Tryptic soy broth + 10% (v/v) human plasma; RPMI + HP – RPMI 1640 + 10% (v/v) human plasma; Lubbock – Bolton broth + 50% (v/v) human plasma + 5% (v/v) sheep red blood cell lysate; RPMI w/o GLU + HP + RBC – RPMI 1640 without glucose + 33% (v/v) human plasma + 5% (v/v) sheep red blood cell lysate; MRSA – methicillin-resistant *Staphylococcus aureus* (ATCC 43300); CA – *Candida albicans* (ATCC 90028); AFG – anidulafungin; CIP – ciprofloxacin; eDNA – extracellular DNA, CFU/ml – colony-forming units/mL. Figure created in BioRender.com.

Summarised procedures are shown in Fig. 7.

## Electronic supplementary material

Below is the link to the electronic supplementary material.

Supplementary Material 1

## Data Availability

The data supporting the findings of this study are available within the article and in the supplementary information. Other data related to this study are available from the corresponding author upon reasonable request.

## References

[1] World Health Organization. *Antimicrobial resistance.* (2023). https://www.who.int/news-room/fact-sheets/detail/antimicrobial-resistance

[2] Singh, S., Singh, S. K., Chowdhury, I. & Singh, R. Understanding the mechanism of bacterial biofilms resistance to antimicrobial agents. *Open. Microbiol. J.* **11**, 53–62 (2017). 28553416 10.2174/1874285801711010053 PMC5427689

[3] Penesyan, A., Paulsen, I. T., Kjelleberg, S. & Gillings, M. R. Three faces of biofilms: a microbial lifestyle, a nascent multicellular organism, and an incubator for diversity. *NPJ Biofilms Microbiomes*. **7**, 80 (2021). 34759294 10.1038/s41522-021-00251-2 PMC8581019

[4] Karygianni, L., Ren, Z., Koo, H. & Thurnheer, T. Biofilm matrixome: extracellular components in structured microbial communities. *Trends Microbiol.* **28** (8), 668–681 (2020). 32663461 10.1016/j.tim.2020.03.016

[5] Berlanga, M. & Guerrero, R. Living together in biofilms: the microbial cell factory and its biotechnological implications. *Microb. Cell. Fact.* **15**, 165 (2016). 27716327 10.1186/s12934-016-0569-5 PMC5045575

[6] Li, X. H. & Lee, J. H. Antibiofilm agents: a new perspective for antimicrobial strategy. *J. Microbiol.* **55** (10), 753–766 (2017). 28956348 10.1007/s12275-017-7274-x

[7] Tytgat, H. L. P., Nobrega, F. L., van der Oost, J. & de Vos, W. M. Bowel biofilms: tipping points between a healthy and compromised gut? *Trends Microbiol.* **27**, 17–25 (2019). 30219265 10.1016/j.tim.2018.08.009

[8] Anju, V. T. et al. Polymicrobial infections and biofilms: clinical significance and eradication strategies. *Antibiot. (Basel)*. **11** (12), 1731 (2022). 10.3390/antibiotics11121731 PMC9774821 36551388

[9] Rao, Y., Shang, W., Yang, Y., Zhou, R. & Rao, X. Fighting mixed-species microbial biofilms with cold atmospheric plasma. *Front. Microbiol.* **11**, 1000 (2020). 32508796 10.3389/fmicb.2020.01000 PMC7251026

[10] Yuan, L. et al. Mixed-species biofilms in the food industry: current knowledge and novel control strategies. *Crit. Rev. Food Sci. Nutr.* **60** (13), 2277–2293 (2020). 31257907 10.1080/10408398.2019.1632790

[11] Carolus, H., Van Dyck, K. & Van Dijck, P. *Candida albicans* and *Staphylococcus* species: a threatening twosome. *Front. Microbiol.* **10**, 2162 (2019). 31620113 10.3389/fmicb.2019.02162 PMC6759544

[12] Kalita, S. et al. Dual delivery of chloramphenicol and essential oil by poly-ε-caprolactone–pluronic nanocapsules to treat MRSA-*Candida* co-infected chronic burn wounds. *Roy Soc. Chem.* **7**, 1749–1758 (2017).

[13] Kong, E. F. et al. Commensal protection of *Staphylococcus aureus* against antimicrobials by *Candida albicans* biofilm matrix. *mBio* **7** (5), e01365–e01316 (2016). 27729510 10.1128/mBio.01365-16 PMC5061872

[14] Gupta, N., Haque, A., Mukhopadhyay, G., Narayan, R. P. & Prasad, R. Interactions between bacteria and Candida in the burn wound. *Burns* **31** (3), 375–378 (2005). 15774298 10.1016/j.burns.2004.11.012

[15] Metcalf, D. G. & Bowler, P. G. Biofilm delays wound healing: a review of the evidence. *Burns Trauma.* **1**, 5–12 (2013). 27574616 10.4103/2321-3868.113329 PMC4994495

[16] Percival, S. L. Importance of biofilm formation in surgical infection. *Br. J. Sur*. **104** (2), e85–e94 (2017). 10.1002/bjs.10433 28121033

[17] Guzmán-Soto, I. et al. Mimicking biofilm formation and development: recent progress in *in vitro* and *in vivo* biofilm models. *iScience* **24** (5), 102443 (2021). 34013169 10.1016/j.isci.2021.102443 PMC8113887

[18] Powers, J. H. Antimicrobial drug development – the past, the present, and the future. *Clin. Microbiol. Infect.* **10**, 23–31 (2004). 15522037 10.1111/j.1465-0691.2004.1007.x

[19] Zhang, K., Li, X., Yu, C. & Wang, Y. Promising therapeutic strategies against microbial biofilm challenges. *Front. Cell. Infect. Microbiol.* **10**, 359 (2020). 32850471 10.3389/fcimb.2020.00359 PMC7399198

[20] Vyas, H. K. N., Xia, B. & Mai-Prochnow, A. Clinically relevant *in vitro* biofilm models: a need to mimic and recapitulate the host environment. *Biofilm* **4**, 100069 (2022). 36569981 10.1016/j.bioflm.2022.100069 PMC9782257

[21] Sun, Y., Dowd, S. E., Smith, E., Rhoads, D. D. & Wolcott, R. D. *In vitro* multispecies Lubbock chronic wound biofilm model. *Wound Repair. Regen*. **16** (6), 805–813 (2008). 19128252 10.1111/j.1524-475X.2008.00434.x

[22] Diepoltová, A., Konečná, K., Janďourek, O. & Nachtigal, P. Study of the impact of cultivation conditions and peg surface modification on the *in vitro* biofilm formation of *Staphylococcus aureus* and *Staphylococcus epidermidis* in a system analogous to the Calgary biofilm device. *J. Med. Microbiol.*. 10.1099/jmm.0.001371 (2021). 10.1099/jmm.0.001371 34048334

[23] Konečná, K., Němečková, I., Diepoltová, A., Vejsová, M. & Janďourek, O. The impact of cultivation media on the *in vitro* biofilm biomass production of candida Spp. *Curr. Microbiol.* **78** (5), 2104–2111 (2021). 33765192 10.1007/s00284-021-02452-6

[24] Stepanović, S. et al. Quantification of biofilm in microtiter plates: overview of testing conditions and practical recommendations for assessment of biofilm production by staphylococci. *APMIS* **115** (8), 891–899 (2007). 17696944 10.1111/j.1600-0463.2007.apm_630.x

[25] Wu, H., Moser, C., Wang, H. Z., Høiby, N. & Song, Z. J. Strategies for combating bacterial biofilm infections. *Int. J. Oral Sci.* **1 7**, 1–7 (2015). 25504208 10.1038/ijos.2014.65 PMC4817533

[26] Costa, R. C. et al. Polymicrobial biofilms related to dental implant diseases: unravelling the critical role of extracellular biofilm matrix. *Crit. Rev. Microbiol.* **49** (3), 370–390 (2023). 35584310 10.1080/1040841X.2022.2062219

[27] Zago, C. E. et al. Dynamics of biofilm formation and the interaction between *Candida albicans* and methicillin-susceptible (MSSA) and -resistant *Staphylococcus aureus* (MRSA). *PloS One*, **10**(4), e0123206 (2015). 10.1371/journal.pone.0123206 PMC4395328 25875834

[28] Lade, H. et al. Biofilm formation by *Staphylococcus aureus* clinical isolates is differentially affected by glucose and sodium chloride supplemented culture media. *J. Clin. Med.* **8** (11), 1853 (2019). 31684101 10.3390/jcm8111853 PMC6912320

[29] Leonhard, M., Zatorska, B. & Moser, D. & Schneider-Stickler, B. Growth Media for mixed multispecies oropharyngeal biofilm compositions on silicone. *Biomed Res. Int.* 8051270 (2019). (2019). 10.1155/2019/8051270 PMC6652045 31360725

[30] Pynnonen, M., Stephenson, R. E., Schwartz, K., Hernandez, M. & Boles, B. R. Hemoglobin promotes *Staphylococcus aureus* nasal colonization. *PLoS Pathog* **7**(7), e1002104 (2011). 10.1371/journal.ppat.1002104 PMC3131264 21750673

[31] Weerasekera, M. M. et al. Culture media profoundly affect *Candida albicans* and *Candida tropicalis* growth, adhesion and biofilm development. *Mem. Inst. Oswaldo Cruz*. **111** (11), 697–702 (2016). 27706381 10.1590/0074-02760160294 PMC5125054

[32] Diban, F. et al. Biofilms in chronic wound infections: innovative antimicrobial approaches using the in vitro Lubbock chronic wound biofilm model. *Int. J. Mol. Sci.* **24** (2), 1004 (2023). 36674518 10.3390/ijms24021004 PMC9862456

[33] Hourigan, L. A. et al. Loss of protein, immunoglobulins, and electrolytes in exudates from negative pressure wound therapy. *Nut Clin. Pract.* **25** (5), 510–516 (2010). 10.1177/0884533610379852 20962311

[34] Cardile, A. P. et al. Human plasma enhances the expression of staphylococcal microbial surface components recognizing adhesive matrix molecules promoting biofilm formation and increases antimicrobial tolerance in Vitro. *BMC Res. Notes*. **7**, 457 (2014). 25034276 10.1186/1756-0500-7-457 PMC4110374

[35] Halfman, C. *Laboratory examination of serous fluids*. (2014). http://www.clinbiochemtopics.com/Topics/Fall/SersFlud/

[36] Innes, E., Yiu, H. H. P., McLean, P., Brown, W. & Boyles, M. Simulated biological fluids - a systematic review of their biological relevance and use in relation to inhalation toxicology of particles and fibres. *Crit. Rev. Toxicol.* **51** (3), 217–248 (2021). 33905298 10.1080/10408444.2021.1903386

[37] Löffler, M. W., Schuster, H., Bühler, S. & Beckert, S. Wound fluid in diabetic foot ulceration: more than just an undefined soup? *Int. J. Low Extrem Wounds*. **12** (2), 113–129 (2013). 23771612 10.1177/1534734613489989

[38] Metcalf, D. G. et al. Elevated wound fluid pH correlates with increased risk of wound infection. *Wound Med.* **26**, (2019).

[39] Trengove, N. J., Langton, S. R. & Stacey, M. C. Biochemical analysis of wound fluid from nonhealing and healing chronic leg ulcers. *Wound Repair. Regen*. **4** (2), 234–239 (1996). 17177819 10.1046/j.1524-475X.1996.40211.x

[40] Jung, P. et al. *Candida albicans* adhesion to central venous catheimpactImpact of blood plasma-driven germ tube formation and pathogen-derived adhesins. *Virulence* **11**, 1453–1465 (2020). 33108253 10.1080/21505594.2020.1836902 PMC7595616

[41] Nobile, C. J. & Johnson, A. D. *Candida albicans* biofilms and human disease. *Annu. Rev. Microbiol.* **69**, 71–92 (2015). 26488273 10.1146/annurev-micro-091014-104330 PMC4930275

[42] Dauros-Singorenko, P., Wiles, S. & Swift, S. *Staphylococcus aureus* biofilms and their response to a relevant *in vivo* iron source. *Front. Microbiol.* **11**, 509525 (2020). 33408695 10.3389/fmicb.2020.509525 PMC7779473

[43] Watchaputi, K., Jayasekara, L. A. C. B., Ratanakhanokchai, K. & Soontorngun, N. Inhibition of cell cycle-dependent hyphal and biofilm formation by a novel cytochalasin 19,20epoxycytochalasin Q in *Candida albicans*. *Sci. Rep.* **13**, 9724 (2023). 37322086 10.1038/s41598-023-36191-4 PMC10272203

[44] Zolla, L. Proteomics studies reveal important information on small molecule therapeutics: a case study on plasma proteins. *Drug Discov Today*. **13** (23–24), 1042–1051 (2008). 18973825 10.1016/j.drudis.2008.09.013 PMC7185545

[45] Batoni, G., Martinez-Pomares, L. & Esin, S. Editorial: Immune response to biofilms. *Front. Immunol.* **12**, 696356 (2021). 34163492 10.3389/fimmu.2021.696356 PMC8215378

[46] Marsh, P. D. & Devine, D. A. How is the development of dental biofilms influenced by the host? *J. Clin. Peridontol*. **38** (Suppl 11), 28–35 (2011). 10.1111/j.1600-051X.2010.01673.x 21323701

[47] Briški, F. & Vuković Domanovac, M. Environmental microbiology. *Phys. Sci. Rev.* **2** (11), 20160118 (2017).

[48] Waldrop, R., McLaren, A., Calara, F. & McLemore, R. Biofilm growth has a threshold response to glucose *in vitro*. *Clin. Orthop. Relat. Res.* **472** (11), 3305–3310 (2014). 24599648 10.1007/s11999-014-3538-5 PMC4182383

[49] Afshar, P., Larijani, L. V. & Rouhanizadeh, H. A comparison of conventional rapid methods in diagnosis of superficial and cutaneous mycoses based on KOH, Chicago sky blue 6B and calcofluor white stains. *Iran. J. Microbiol.* **10** (6), 433–440 (2018). 30873272 PMC6414738

[50] Van Ende, M., Wijnants, S. & Van Dijck, P. Sugar sensing and signaling in *Candida albicans* and *Candida Glabrata*. *Front. Microbiol.* **10**, 99 (2019). 30761119 10.3389/fmicb.2019.00099 PMC6363656

[51] Rosenberg, M., Azevedo, N. F. & Ivask, A. Propidium iodide staining underestimates viability of adherent bacterial cells. *Sci. Rep.* **9** (1), 6483 (2019). 31019274 10.1038/s41598-019-42906-3 PMC6482146

[52] Harriott, M. M. & Noverr, M. C. Importance of Candida-bacterial polymicrobial biofilms in disease. *Trends Microbiol.* **19** (11), 557–563 (2011). 21855346 10.1016/j.tim.2011.07.004 PMC3205277

[53] Mishra, S. et al. Therapeutic strategies against biofilm infections. *Life (Basel)*. **13** (1), 172 (2023). 36676121 10.3390/life13010172 PMC9866932

[54] Liesenborghs, L., Verhamme, P. & Vanassche, T. *Staphylococcus aureus*, master manipulator of the human hemostatic system. *J. Thromb. Haemost*. **16** (3), 441–454 (2018). 29251820 10.1111/jth.13928

[55] Stewart, P. S. & Franklin, M. J. Physiological heterogeneity in biofilms. *Nat. Rev. Microbiol.* **6** (3), 199–210 (2008). 18264116 10.1038/nrmicro1838

[56] Christensen, G. D. et al. Adherence of coagulase-negative staphylococci to plastic tissue culture plates: a quantitative model for the adherence of staphylococci to medical devices. *J. Clin. Microbiol.* **22** (6), 996–1006 (1985). 3905855 10.1128/jcm.22.6.996-1006.1985 PMC271866

[57] European Committee for Antimicrobial Susceptibility Testing. Determination of minimum inhibitory concentrations (MICs) of antibacterial agents by broth dilution. *Clin. Microbiol. Infect.* **9** (8), 1–7 (2003).12691538

[58] European Committee for Antimicrobial Susceptibility Testing. *EUCAST definitive document E.DEF 7.3.1*. (2017). https://www.eucast.org/fileadmin/src/media/PDFs/EUCAST_files/AFST/Files/EUCAST_E_Def_7_3_1_Yeast_testing__definitive.pdf

