# Supplementary material for: The appropriate nutrient conditions for methicillin-resistant Staphylococcus aureus and Candida albicans dual-species biofilm formation in vitro
Source: Sci Rep. 2025 Jan 2;15:183. doi: 10.1038/s41598-024-83745-1 (PMC11696109; doi:10.1038/s41598-024-83745-1)
Supplement: Supplementary file 1 — Supplementary Material 1 [file 41598_2024_83745_MOESM1_ESM.pdf]

The appropriate nutrient conditions for methicillin-resistant *Staphylococcus aureus* and *Candida albicans* dual-species biofilm formation *in vitro*

Pavlína Vávrová, Ondřej Janďourek, Adéla Diepoltová, Petr Nachtigal, Klára Konečná*

Charles University, Faculty of Pharmacy in Hradec Králové, Department of Biological and Medical Sciences, Hradec Králové, Czech Republic

*Address correspondence to Klára Konečná, konecna@faf.cuni.cz
Charles University, Faculty of Pharmacy in Hradec Králové, Department of Biological and Medical Sciences, Zborovská 2089, 500 03, Hradec Králové, Czech Republic, Tel: + (420) 495 067 366, ORCID:0000-0001-5670-7767

Supplementary information

Table S1: Comparison of the basic composition of the four cultivation media chosen for methicillin-resistant *Staphylococcus aureus* and *Candida albicans* dual-species biofilm formation *in vitro*

Cultivation medium composition	Medium shortcut	Proteins/peptides/ amino acids (g/L)	Glucose (g/L)	Final pH
Tryptic soy broth + 10 % (v/v) human plasma	TSB+HP	25.00-28.00	2.60-2.64	7.3±0.2
RPMI 1640 + 10 % (v/v) human plasma	RPMI+HP	6.00-9.00	2.10-2.14	7.4±0.2
Bolton broth + 50 % (v/v) human plasma + 5 % (v/v) sheep red blood cell lysate	Lubbock	62.50-77.50	0.59-0.81	7.4±0.2
RPMI 1640 without glucose + 33 % (v/v) human plasma +	RPMI w/o GLU+HP+RBC	34.00-43.90	0.42-0.57	7.2±0.2

5 % (v/v) sheep red blood cell lysate				
Wound exudate*		20.00-38.00	≤0.85	7.2±0.2

*Individual values are compilated [34-39]

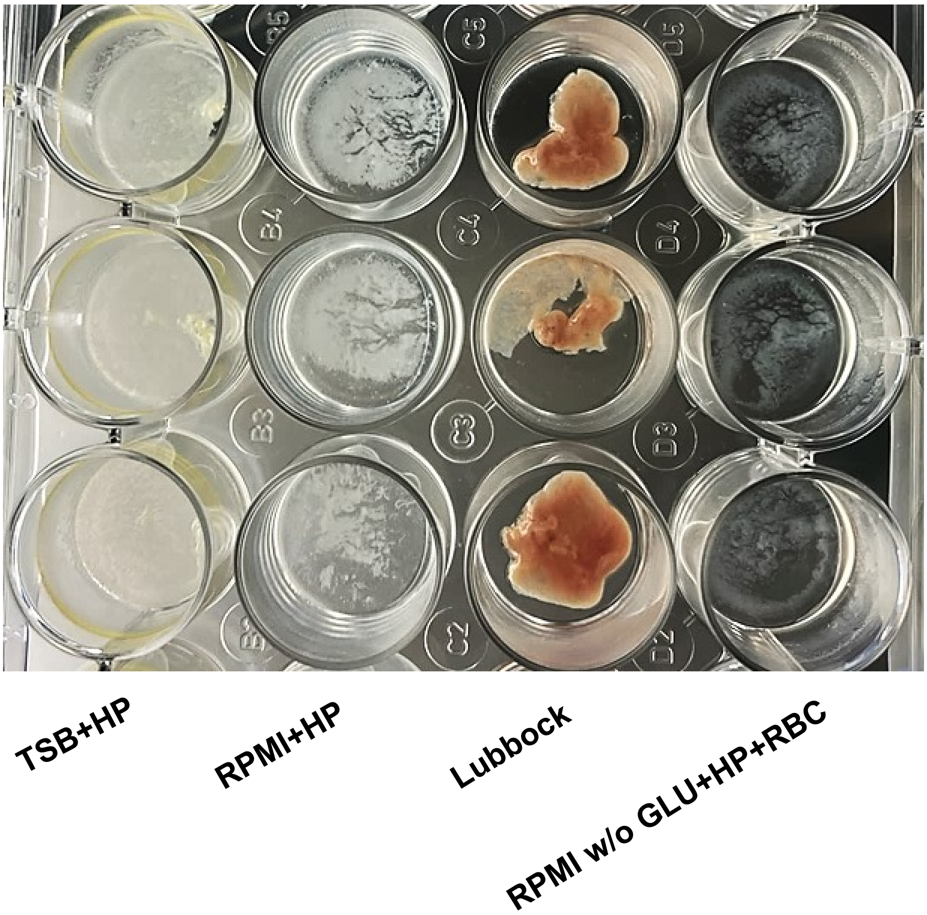

Fig. S1: Visual inspection of dual-species biofilms

Methicillin-resistant *Staphylococcus aureus* (ATCC 43300) and *Candida albicans* (ATCC 90028) dual-species biofilm biomasses were cultivated *in vitro* in 24-well culture plate for 24 h in different cultivation media: TSB+HP – Tryptic soy broth + 10 % (v/v) human plasma, RPMI+HP – RPMI 1640 + 10 % (v/v) human plasma, Lubbock – Bolton broth + 50 % (v/v) human plasma + 5 % (v/v) sheep red blood cell lysate, RPMI

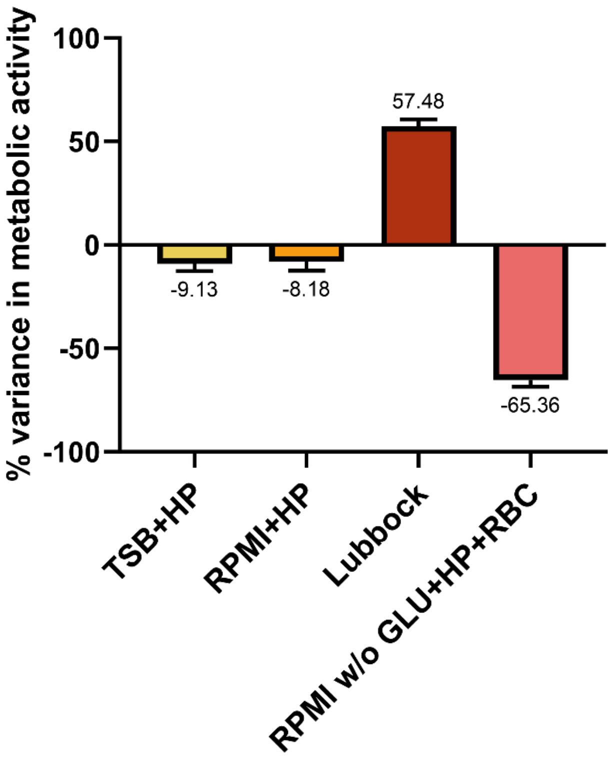

w/o GLU+HP+RBC – RPMI 1640 without glucose + 33 % (v/v) human plasma + 5 % (v/v) sheep red blood cell lysate

Fig. S2: Comparison of withstanding hostile conditions of dual-species biofilms

24-hours-old methicillin-resistant *Staphylococcus aureus* (ATCC 43300) and *Candida albicans* (ATCC 90028) dual-species biofilms formed in selected cultivation media: TSB+HP – Tryptic soy broth + 10 % (v/v) human plasma, RPMI+HP – RPMI 1640 + 10 % (v/v) human plasma, Lubbock – Bolton broth + 50 % (v/v) human plasma + 5 % (v/v) sheep red blood cell lysate, RPMI w/o GLU+HP+RBC – RPMI 1640 without glucose + 33 % (v/v) human plasma + 5 % (v/v) sheep red blood cell lysate were exposed for 24 h to the mixture of selected antimicrobial drugs (ciprofloxacin and anidulafungin). The values represent the mean ± SEM. % variance in metabolic activity – the metabolic activity of biofilm-forming microbial participants after antimicrobial

drugs exposure related to the metabolic activity of unexposed biofilm-forming microbial participants before drugs exposition (100 %).

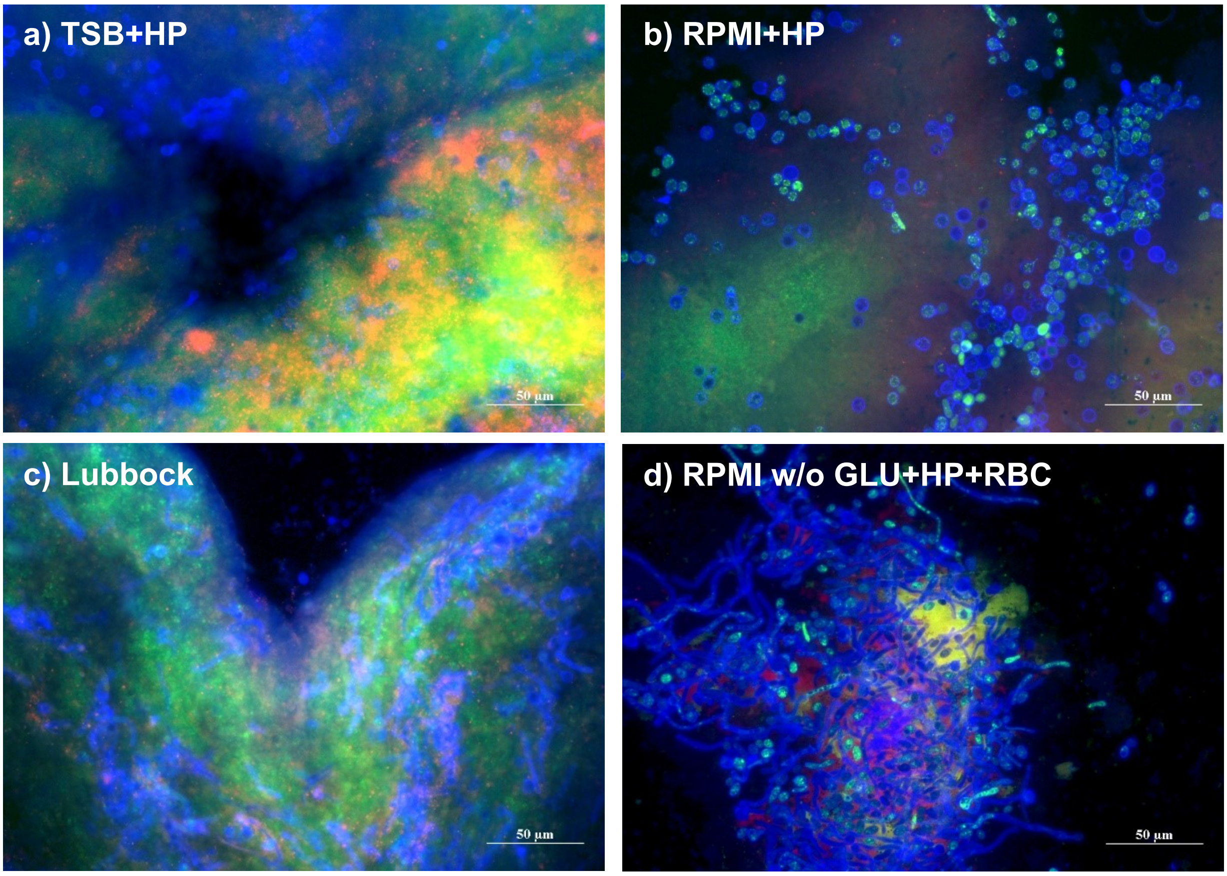

Fig. S3: Visualization of dual-species biofilms by fluorescent microscopy (SYTO 9 + PI + CW)

Dual-species methicillin-resistant *Staphylococcus aureus* (ATCC 43300) and *Candida albicans* (ATCC 90028) biofilm consortia were formed 24 hours in the four selected cultivation media: a) TSB+HP – Tryptic soy broth + 10 % (v/v) human plasma; b) RPMI+HP – RPMI 1640 + 10 % (v/v) human plasma; c) Lubbock – Bolton broth + 50 % (v/v) human plasma + 5 % (v/v) sheep red blood cell lysate; d) RPMI w/o GLU+HP+RBC – RPMI 1640 without glucose + 33 % (v/v) human plasma + 5 % (v/v) sheep red blood cell lysate. Triple-combination of fluorescent dyes, Calcofluor White, SYTO 9, and propidium iodide were employed for microbial cells (blue yeast cells,

green living bacterial cells, red dead bacterial/yeast cells) and biofilm matrix (blue, green, or red stained unbounded, dispersed mass) visualization. The scale bar corresponds to 50 μm.

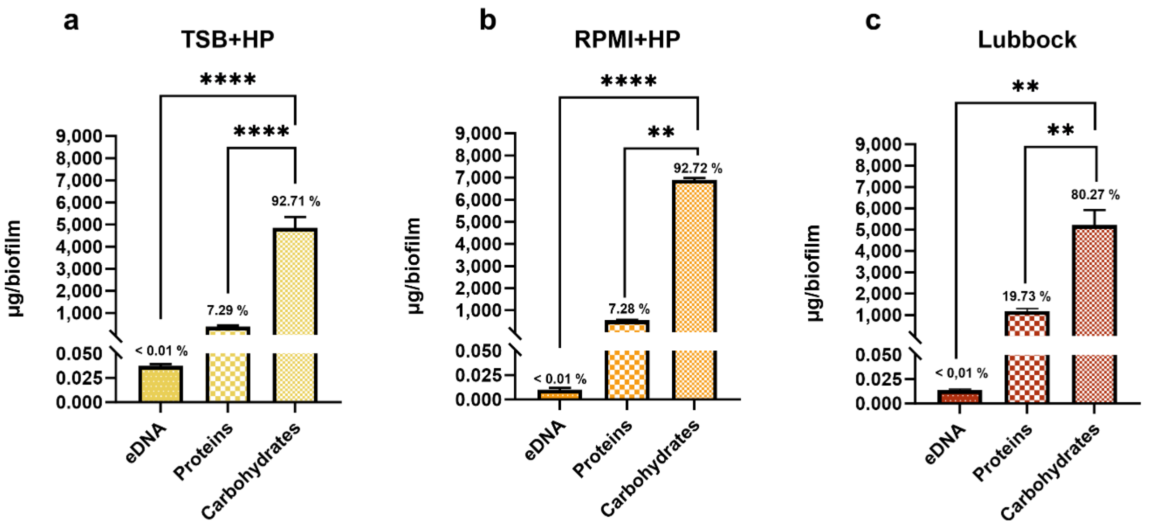

Fig. S4: Representation of the key biofilm matrix biomolecules in dual-species biofilms

Methicillin-resistant *Staphylococcus aureus* (ATCC 43300) and *Candida albicans* (ATCC 90028) dual-species biofilm consortia were formed for 24 hours in three selected cultivation media a) TSB+HP – Tryptic soy broth + 10 % (v/v) human plasma; b) RPMI+HP – RPMI 1640 + 10 % (v/v) human plasma; c) Lubbock – Bolton broth + 50 % (v/v) human plasma + 5 % (v/v) sheep red blood cell lysate. The values represent the mean ± SEM. The percentages above the columns express the weight amount related to total biomolecule mass (sum of proteins, carbohydrates, and extracellular DNA (eDNA) masses). To compare the dominance of carbohydrates with proteins and eDNA, data were analysed using t-test, and a *p*-value < 0.05 was accepted as statistically significant.
